# Optimal Convergence Analysis of Two-Level Nonconforming Finite Element Iterative Methods for 2D/3D MHD Equations

**DOI:** 10.3390/e24050587

**Published:** 2022-04-22

**Authors:** Haiyan Su, Jiali Xu, Xinlong Feng

**Affiliations:** College of Mathematics and System Sciences, Xinjiang University, Urumqi 830046, China; shymath@126.com (H.S.); xujiali2015@126.com (J.X.)

**Keywords:** incompressible MHD equations, nonconforming finite element, two-level method, stability, error estimate

## Abstract

Several two-level iterative methods based on nonconforming finite element methods are applied for solving numerically the 2D/3D stationary incompressible MHD equations under different uniqueness conditions. These two-level algorithms are motivated by applying the *m* iterations on a coarse grid and correction once on a fine grid. A one-level Oseen iterative method on a fine mesh is further studied under a weak uniqueness condition. Moreover, the stability and error estimate are rigorously carried out, which prove that the proposed methods are stable and effective. Finally, some numerical examples corroborate the effectiveness of our theoretical analysis and the proposed methods.

## 1. Introduction

Magnetohydrodynamics (MHD) describes the interaction of electrically conductive fluids with electromagnetic fields. The governing equations describing the MHD system is a strong coupling nonlinear system coupled with the Navier–Stokes equations and Maxwell equations. Magnetohydrodynamics has become widespread in such areas of astrophysics, controlled thermonuclear reactions and industry. For the study of the MHD problem, a large amount of research and analysis have been carried out in recent decades. The well-posedness of weak form solution of MHD equations can be guaranteed in [1,2]. With regard to theoretical analysis, the regularity, long-time behaviors of solution of MHD problems and the error estimation of FEM are studied in [3,4]. We can refer to [5,6,7,8,9] and their references for many Galerkin finite element methods (FEM) analysis and studied on the MHD system. For the MHD problem, a series of one-level iteration methods and their error estimation are studied in [10], and some coupled type iteration methods are designed and discussed in [11] on a general Lipschitz domain.

The present paper mainly focuses on the study of nonconforming finite element. Low-order nonconforming FEM has the advantages over the conforming FEM in terms of simplicity and small support sets of basis functions. For the Stokes and Navier–Stokes equation, the nonconforming FEM are studied in [12,13], in which the discretization of velocity space uses the nonconforming element, and the discretization of pressure space uses the piecewise constant element. In addition, a nonconforming FEM are also proposed in [14,15,16], which differs from the discrete pressure space using a piecewise linear element. Due to the limitation of inf-sup condition, it has the advantage of simple structure and has been well applied in solving various problems. For instance, an Oseen iterative algorithm for the conduction-convection equations with nonconforming FEM is carefully studied in [17]. In addition, the low-order nonconforming FEM is used to solve 3D MHD system, then they make deep and systematic analysis and research in [18,19].

In order to raise the efficiency of computation, much work has sought to answer two-level methods for solving the nonlinear problems with conforming FEM. For the Navier–Stokes equations, some two-grid schemes and their error estimation are presented in [20,21,22,23,24]. Regarding the two-level methods approach to discussing the MHD problems, we can refer to the literature [25,26,27]. Furthermore, the MHD problem is studied well enough by a two-level Newton method at a small magnetic Reynolds number and a two-level method under the hypotheses of a small data in [28,29]. In addition, some two-level iterative methods are used to solve the MHD problem, then rigorous systematic analysis and numerical tests are carried out in [30].

In our previous work, three linearized and one level nonconforming discretization for the 2D/3D MHD equations were proposed in [31]. In order to solve it more efficiently and to make our work more completely, the algorithms we study are looking to link two-level iterative methods with the nonconforming FEM to solve the MHD problem. The study is based on approximating velocity space by the nonconforming element, and the conforming linear elements are used in the discretization of magnetic field and pressure space. We analyze the error estimates of three classical one-level iterative methods under different uniqueness conditions. Then, more comprehensive and diverse two-level iterative methods based on the iterative solution firstly calculated by Stokes, Newton and Oseen iterative methods on a coarse grid and then the correction solution calculated by Stokes, Newton and Oseen corrections on a fine grid are proposed. Moreover, we perform a systematic and in-depth analysis of our proposed methods under the different strong uniqueness conditions. Finally, several numerical texts are executed and the accuracy of the proposed methods are proved.

The following describes the components of this article. The mathematical setting of the MHD equations is introduced and the nonconforming FEM is proposed in Section 2 and Section 3. In Section 4, three classical one-level iterative methods and error analysis results are given. In Section 5, we put forward some two-level algorithms and deduce more comprehensively theoretical analysis. In Section 6, several numerical simulations are tested to verify the accuracy of the previous results. In the last part, the summary and prospects of the paper are given.

In this next paper, C represents a real constant, which represents different values in different cases and is independent of the coefficients of the system equations, the grid sizes *h* and *H*. Notations without special interpretation are used for their usual meaning.

## 2. Preliminaries

Let Ω be a bounded convex region in $R^{d}$(d=2,3) with boundary ∂Ω, to study the 2D/3D stationary incompressible MHD problem, which is modeled as listed below: find the velocity u, magnetic field b and pressure *p* such that

(1) $$\begin{array}{c} \left\{\begin{array}{c} -R_{e}^{-1}\Delta u+u\cdot \nabla u+\nabla p-S_{c}\left(\nabla \times b\right)\times b=f,\;\mathrm{in}\;\Omega ,\\ R_{m}^{-1}S_{c}\nabla \times \left(\nabla \times b\right)-S_{c}\nabla \times \left(u\times b\right)=g,\;\mathrm{in}\;\Omega ,\\ \nabla \cdot u=0,\;\mathrm{in}\;\Omega ,\\ \nabla \cdot b=0,\;\mathrm{in}\;\Omega ,\\ u=0,\;b\cdot n=0,\;(\nabla \times b)\times n=0,\;\mathrm{on}\;\partial \Omega , \end{array}\right. \end{array}$$

where $f\in H^{-1}{\left(\Omega \right)}^{d},g\in L^{2}{\left(\Omega \right)}^{d}$ denote prescribed body terms, and n represents outward unit normal vector. The nonlinear system includes three different coefficients: hydrodynamic Reynolds number $R_{e}$, magnetic Reynolds number $R_{m}$, and coupling coefficient $S_{c}$. It should still be noted out that the actual physically meaningful induction equation corresponds only to the special case g = 0.

For convenience, the function spaces are shown below:

$$\begin{array}{c} \begin{array}{c} L_{0}^{2}\left(\Omega \right)=\left\{r\in L^{2}\left(\Omega \right):\int_{\Omega }rdx=0\right\},\\ H_{0}^{1}\left(\Omega \right)=\left\{e\in H^{1}{\left(\Omega \right)}^{d}:{\left.e\right|}_{\partial \Omega }=0\right\},\\ H_{n}^{1}\left(\Omega \right)=\left\{e\in H^{1}{\left(\Omega \right)}^{d}:{\left.e\cdot n\right|}_{\partial \Omega }=0\right\}. \end{array} \end{array}$$

Combining the above spaces, the weak form of (1) is equivalent to finding $\left(u,b\right)\in H_{0}^{1}\left(\Omega \right)\times H_{n}^{1}\left(\Omega \right)$, $p\in L_{0}^{2}\left(\Omega \right)$ such that, for all $\left(\kappa ,D\right)\in H_{0}^{1}\left(\Omega \right)\times H_{n}^{1}\left(\Omega \right),r\in L_{0}^{2}\left(\Omega \right)$,

(2) $$\begin{array}{c} \left\{\begin{array}{c} A_{0}\left(\left(u,b\right),\left(\kappa ,D\right)\right)+A_{1}\left(\left(u,b\right),\left(u,b\right),\left(\kappa ,D\right)\right)-b\left(\left(\kappa ,D\right),p\right)=<F,\left(\kappa ,D\right)>,\\ b((u,b),r)=0, \end{array}\right. \end{array}$$

with the bilinear and trilinear forms settings below

$$\begin{array}{c} \begin{array}{c} A_{0}\left(\left(e,E\right),\left(\kappa ,D\right)\right):=a_{0}\left(e,\kappa \right)+b_{0}\left(E,D\right),\\ A_{1}\left(\left(u,b\right),\left(e,E\right),\left(\kappa ,D\right)\right):=a_{1}\left(u,e,\kappa \right)-c\left(E,b,\kappa \right)+c\left(D,b,e\right),\\ a_{0}\left(e,\kappa \right):=R_{e}^{-1}\left(\nabla e,\nabla \kappa \right),\;b_{0}\left(E,D\right):=R_{m}^{-1}S_{c}\left(\nabla \times E,\nabla \times D\right)+R_{m}^{-1}S_{c}\left(\nabla \cdot E,\nabla \cdot D\right),\\ a_{1}\left(u,e,\kappa \right):=\frac{1}{2}\left(u\cdot \nabla e,\kappa \right)-\frac{1}{2}\left(u\cdot \nabla \kappa ,e\right),\;c\left(E,b,\kappa \right):=S_{c}\left(\nabla \times E\times b,\kappa \right),\\ b((\kappa ,D),r):=(\nabla \cdot \kappa ,r),\;<F,(\kappa ,D)>:=<f,\kappa >+(g,D). \end{array} \end{array}$$

Furthermore, the norms are written by

$$\begin{array}{c} \begin{array}{cc} & {∥\left(\kappa ,D\right)∥}_{i}:={\left({∥\kappa ∥}_{i}^{2}+{∥D∥}_{i}^{2}\right)}^{\frac{1}{2}},\;\forall \kappa \in H^{i}\left(\Omega \right)\cap H_{0}^{1}\left(\Omega \right),D\in H^{i}\left(\Omega \right)\cap H_{n}^{1}\left(\Omega \right)\left(i=0,1,2\right).\\ & {∥F∥}_{-1}:=\underset{0\neq \left(\kappa ,D\right)\in H_{0}^{1}\left(\Omega \right)\times H_{n}^{1}\left(\Omega \right)}{\sup}\frac{<F,(\kappa ,D)>}{{∥\left(\kappa ,D\right)∥}_{1}}{,\;∥F∥}_{0}:={(∥f∥}_{0}+{∥g∥}_{0}{)}^{\frac{1}{2}}. \end{array} \end{array}$$

Next, we introduce some Sobolev inequalities [10,26],

$$\begin{array}{c} \begin{array}{cc} & {∥e∥}_{L^{4}}\leq \lambda_{0}{∥e∥}_{1},\;\forall e\in H_{0}^{1}\left(\Omega \right),\\ & {∥e∥}_{L^{3}}\leq C{∥e∥}_{0}^{\frac{1}{2}}{∥e∥}_{1}^{\frac{1}{2}},\;\forall e\in H_{0}^{1}\left(\Omega \right),\\ & {∥e∥}_{L^{\infty }}\leq C{∥e∥}_{1}^{\frac{1}{2}}{∥e∥}_{2}^{\frac{1}{2}},\;\forall e\in H^{2}\left(\Omega \right)\cap H_{0}^{1}\left(\Omega \right). \end{array} \end{array}$$

According to the above inequality, we can obtain the properties from [10,11,18],

$$\begin{array}{c} \begin{array}{c} a_{1}\left(u,e,\kappa \right)=-a_{1}\left(u,\kappa ,e\right),\;\left|a_{1}\left(u,e,\kappa \right)\right|\leq \lambda_{0}^{2}{∥u∥}_{1}{∥e∥}_{1}{∥\kappa ∥}_{1},\;\forall u,e,\kappa \in H_{0}^{1}\left(\Omega \right). \end{array} \end{array}$$

In addition, we have the following properties from [10,11,18]: for all $u,\kappa ,e\in H_{0}^{1}\left(\Omega \right)$, and $b,D,E\in H_{n}^{1}\left(\Omega \right)$,

$$\begin{array}{c} \begin{array}{c} A_{1}\left(\left(u,b\right),\left(\kappa ,D\right),\left(\kappa ,D\right)\right)=0,\\ A_{0}\left(\left(u,b\right),\left(\kappa ,D\right)\right)\leq \max\left\{R_{e}^{-1},\left(2+d\right)R_{m}^{-1}S_{c}\right\}{∥\left(u,b\right)∥}_{1}{∥\left(\kappa ,D\right)∥}_{1},\\ A_{0}\left(\left(\kappa ,D\right),\left(\kappa ,D\right)\right)\geq \min\left\{R_{e}^{-1},\lambda_{1}R_{m}^{-1}S_{c}\right\}{∥\left(\kappa ,D\right)∥}_{1}^{2},\\ A_{1}\left(\left(u,b\right),\left(\kappa ,D\right),\left(e,E\right)\right)\leq \sqrt{2}\lambda_{0}^{2}\max\left\{1,\sqrt{2}S_{c}\right\}{∥\left(u,b\right)∥}_{1}{∥\left(\kappa ,D\right)∥}_{1}{∥\left(e,E\right)∥}_{1}, \end{array} \end{array}$$

where $\lambda_{1}$ is the constant from

$$\begin{array}{c} {∥\nabla \times D∥}_{0}^{2}+{∥\nabla \cdot D∥}_{0}^{2}\geq \lambda_{1}{∥D∥}_{1}^{2},\;\forall D\in H_{n}^{1}\left(\Omega \right). \end{array}$$

Finally, to analyze the error estimates in the following sections, we can obtain the important theorem as follows [2,11,18]:

**Theorem** **1.** *Suppose that $\frac{\sqrt{2}\lambda_{0}^{2}\max\left\{1,\sqrt{2}S_{c}\right\}{∥F∥}_{-1}}{{\left(\min\left\{R_{e}^{-1},\lambda_{1}R_{m}^{-1}S_{c}\right\}\right)}^{2}}<1$, (2) is well-posed and the unique solution (u,b) satisfies:*

(3) $$\begin{array}{c} {∥\left(u,b\right)∥}_{1}\leq \frac{{∥F∥}_{-1}}{\min\left\{R_{e}^{-1},\lambda_{1}R_{m}^{-1}S_{c}\right\}}. \end{array}$$

To obtain the $H^{2}$ stability of the solution to (2), we give the following assumption where the domain Ω satisfies regular properties as follows.

**Assumption** **1.** *First, the steady Stokes problem is introduced as follows:*

$$\begin{array}{c} {-\Delta \kappa +\nabla r=f,\;\nabla \cdot \kappa =0,\;\mathrm{in}\;\Omega ,\;\kappa |}_{\partial \Omega }=0, \end{array}$$

*and the unique solution (κ,r) satisfies*

$$\begin{array}{c} {∥\kappa ∥}_{2}+{∥r∥}_{1}\leq C{∥f∥}_{0}. \end{array}$$

*Then, we introduce Maxwell’s equations*

$$\begin{array}{c} \nabla \times (\nabla \times D)=g,\;\nabla \cdot D=0\;\mathrm{in}\;\Omega ,\;\nabla \times D\times n=0,\;D\cdot n=0,\;\mathrm{on}\;\partial \Omega . \end{array}$$

*Similarly, the unique solution D satisfies*

$$\begin{array}{c} {∥D∥}_{2}\leq C{∥g∥}_{0}. \end{array}$$

**Remark** **1.** *If* Ω *is a convex polygon or polyhedron, or if ∂Ω is of $C^{2}$, the conclusion is that the assumption is tenable [32,33].*

**Theorem** **2.** *If $f,g\in L^{2}{\left(\Omega \right)}^{d}$, $\frac{\sqrt{2}\lambda_{0}^{2}\max\left\{1,\sqrt{2}S_{c}\right\}{∥F∥}_{-1}}{{\left(\min\left\{R_{e}^{-1},\lambda_{1}R_{m}^{-1}S_{c}\right\}\right)}^{2}}<1$ and Assumption 1 are true, the solution ((u,b),p) satisfies*

(4) $$\begin{array}{c} {∥\left(u,b\right)∥}_{2}+{∥p∥}_{1}\leq C{∥F∥}_{0}. \end{array}$$

## 3. Nonconforming Discretization

Here, we consider the regular triangulation $T_{\mu }=\left\{K\right\}$ that partitions the domain Ω into triangles or quadrangles. Here, we defined the positive parameter $\mu =\max_{K\in T_{\mu }}$$\left\{\mu_{K}:\mu_{K}=\mathrm{diam}\left(K\right)\right\}$, the boundary edge $\Gamma_{j}=\partial K_{j}\cap \partial \Omega$ and the interior boundary $\Gamma_{jk}=\Gamma_{kj}=\partial K_{j}\cap \partial K_{k}$. Denote the centers of $\Gamma_{j}$ and $\Gamma_{jk}$ by $\upsilon_{j}$ and $\upsilon_{jk}$, respectively.

This is followed by the discrete spaces as shown below, in which the discretization of velocity space is approximated by a nonconforming element, and the discretization of pressure and magnetic field space is approximated by a conforming linear element.

$$\begin{array}{cc} & X_{1\mu }=\left\{e_{\mu }\in L^{2}{\left(\Omega \right)}^{d}:{\left.e_{\mu }\right|}_{K}\in P_{1}{\left(K\right)}^{d},\forall K\in T_{\mu };e_{\mu }\left(\upsilon_{jk}\right)=e_{\mu }\left(\upsilon_{kj}\right),e_{\mu }\left(\upsilon_{j}\right)=0,\forall j,k\right\},\\ & M_{\mu }=\left\{r_{\mu }\in H^{1}\left(\Omega \right)\cap L_{0}^{2}\left(\Omega \right):{\left.r_{\mu }\right|}_{K}\in P_{1}\left(K\right),\forall K\in T_{\mu }\right\},\\ & X_{2\mu }=\left\{D_{\mu }\in H_{n}^{1}\left(\Omega \right):{\left.D_{\mu }\right|}_{K}\in P_{1}{\left(K\right)}^{d},D_{\mu }{\cdot n|}_{\partial \Omega }=0,\forall K\in T_{\mu }\right\}, \end{array}$$

where $P_{1}\left(K\right)$ stands for the continuous piecewise polynomial space. In fact, the finite element pair $\left(X_{1\mu },M_{\mu }\right)$ we studied in this paper satisfies the inf-sup condition, which has been rigorously proven in [34].

The finite element spaces $X_{1\mu }$, $X_{2\mu }$ and $M_{\mu }$ satisfy the interpolation theory: for any $\left(\left(\kappa ,D\right),r\right)\in \left(H_{0}^{1}\left(\Omega \right)\cap H^{2}\left(\Omega \right)\right)\times \left(H_{n}^{1}\left(\Omega \right)\cap H^{2}\left(\Omega \right)\right)\times \left(L_{0}^{2}\left(\Omega \right)\cap H^{1}\left(\Omega \right)\right)$, there exist three approximations $((\kappa_{I}$, $D_{I})$, $r_{I})\in X_{1\mu }\times X_{2\mu }\times M_{\mu }$ such that [13,19],

(5) $$\begin{array}{c} \begin{array}{c} ∥\left(\kappa -\kappa_{I},D-D_{I}\right){∥}_{1}+∥(r-r_{I}{∥}_{0}\leq {c\mu (∥\kappa ∥}_{2}+{∥D∥}_{2}+{∥r∥}_{1}). \end{array} \end{array}$$

To give the discrete variational form of (2), we introduce the following compatibility conditions [14,15]:

$$\begin{array}{c} \int_{\Gamma_{jk}}\left[e_{\mu }\right]ds=0,\;\;\int_{\Gamma_{j}}e_{\mu }ds=0, \end{array}$$

where $\left[e_{\mu }\right]=e_{\mu }{{|}_{\Gamma_{jk}}-e_{\mu }|}_{\Gamma_{kj}}$, representing $e_{\mu }$ through an interface $\Gamma_{jk}$.

Combining the above spaces and compatibility conditions, the discrete variational form of (2) is recast: find $\left(u_{\mu },b_{\mu }\right)$$\in X_{1\mu }\times X_{2\mu },p_{\mu }\in M_{\mu }$ such that, for all $\left(\kappa_{\mu },D_{\mu }\right)\in X_{1\mu }\times X_{2\mu },r_{\mu }\in M_{\mu }$,

(6) $$\begin{array}{c} \left\{\begin{array}{c} A_{0}\left(\left(u_{\mu },b_{\mu }\right),\left(\kappa_{\mu },D_{\mu }\right)\right)+A_{1}\left(\left(u_{\mu },b_{\mu }\right),\left(u_{\mu },b_{\mu }\right),\left(\kappa_{\mu },D_{\mu }\right)\right)-b\left(\left(\kappa_{\mu },D_{\mu }\right),p_{\mu }\right)\\ \;\;=<F,(\kappa_{\mu },D_{\mu })>,\\ b(\left(u_{\mu },b_{\mu }\right),r_{\mu })=0. \end{array}\right. \end{array}$$

For all $\kappa_{\mu }\in X_{1\mu },D_{\mu }\in X_{2\mu }$, we define

$$\begin{array}{c} \begin{array}{cc} & {∥\kappa_{\mu }∥}_{0}:={\left(\sum_{K\in T_{\mu }}{∥\kappa_{\mu }∥}_{0,K}^{2}\right)}^{1/2},\;{∥\kappa_{\mu }∥}_{1\mu }:={\left(\sum_{K\in T_{\mu }}{\left|\kappa_{\mu }\right|}_{1,K}^{2}\right)}^{1/2},\\ & {∥\left(\kappa_{\mu },D_{\mu }\right)∥}_{1}:={\left({∥\kappa_{\mu }∥}_{1\mu }^{2}+{∥D_{\mu }∥}_{1\mu }^{2}\right)}^{1/2},\;{∥F∥}_{*}:=\;\underset{0\neq \left(\kappa_{\mu },D_{\mu }\right)\in X_{1\mu }\times X_{2\mu }}{\sup}\frac{<F,\left(\kappa_{\mu },D_{\mu }\right)>}{{∥\left(\kappa_{\mu },D_{\mu }\right)∥}_{1}}. \end{array} \end{array}$$

**Lemma** **1.** *The bilinear and trilinear terms satisfy the following properties:*

(7) $$\begin{array}{c} \begin{array}{cc} & \left|a_{1}\left(u_{\mu },e_{\mu },\kappa_{\mu }\right)\right|\leq N_{1}{∥u_{\mu }∥}_{1\mu }{∥e_{\mu }∥}_{1\mu }{∥\kappa_{\mu }∥}_{1\mu },\\ & \left|c\left(E_{\mu },b_{\mu },\kappa_{\mu }\right)\right|\leq N_{2}{∥b_{\mu }∥}_{1\mu }{∥\kappa_{\mu }∥}_{1\mu }{∥E_{\mu }∥}_{1\mu },\\ & A_{0}\left(\left(u_{\mu },b_{\mu }\right),\left(\kappa_{\mu },D_{\mu }\right)\right)\leq \max\left\{R_{e}^{-1},\left(2+d\right)R_{m}^{-1}S_{c}\right\}∥\left(u_{\mu },b_{\mu }\right){∥}_{1}{∥\left(\kappa_{\mu },D_{\mu }\right)∥}_{1},\\ & A_{0}\left(\left(u_{\mu },b_{\mu }\right),\left(u_{\mu },b_{\mu }\right)\right)\geq \min\left\{R_{e}^{-1},\lambda_{1}R_{m}^{-1}S_{c}\right\}{∥\left(u_{\mu },b_{\mu }\right)∥}_{1}^{2},\\ & A_{1}\left(\left(u_{\mu },b_{\mu }\right),\left(\kappa_{\mu },D_{\mu }\right),\left(e_{\mu },E_{\mu }\right)\right)\\ & \leq \sqrt{2}\max\left\{N_{1},N_{2}\right\}∥\left(u_{\mu },b_{\mu }\right){∥}_{1}∥\left(\kappa_{\mu },D_{\mu }\right){∥}_{1}{∥\left(e_{\mu },E_{\mu }\right)∥}_{1}, \end{array} \end{array}$$

*where $N_{1}={\left(\gamma_{2}\right)}^{2}$, $N_{2}=\sqrt{2}\lambda_{0}\gamma_{2}S_{c}$. $\gamma_{2}$ is the constant from the following discrete imbedding inequality [35,36],*

(8) $$\begin{array}{c} ∥e_{\mu }{∥}_{L^{2m}}\leq \gamma_{m}{∥e_{\mu }∥}_{1\mu },\;\forall e_{\mu }\in X_{1\mu },m=1,2,\cdots . \end{array}$$

**Lemma** **2.** *Here are two important properties of the trilinear forms [27]:*

(9) $$\begin{array}{c} \begin{array}{cc} & A_{1}\left(\left(u_{\mu },b_{\mu }\right),\left(\kappa ,D\right),\left(e_{\mu },E_{\mu }\right)\right)\leq C∥\left(u_{\mu },b_{\mu }\right){∥}_{0}∥\left(\kappa ,D\right){∥}_{2}{∥\left(e_{\mu },E_{\mu }\right)∥}_{1},\\ & \forall \left(u_{\mu },b_{\mu }\right)\in L^{2}{\left(\Omega \right)}^{d}\times L^{2}{\left(\Omega \right)}^{d},\left(\kappa ,D\right)\in H^{2}{\left(\Omega \right)}^{d}\times H^{2}{\left(\Omega \right)}^{d},\left(e_{\mu },E_{\mu }\right)\in X_{1\mu }\times X_{2\mu },\\ & A_{1}\left(\left(u,b\right),\left(\kappa_{\mu },D_{\mu }\right),\left(e_{\mu },E_{\mu }\right)\right)\leq C∥\left(u,b\right){∥}_{2}∥\left(\kappa_{\mu },D_{\mu }\right){∥}_{0}{∥\left(e_{\mu },E_{\mu }\right)∥}_{1},\\ & \forall \left(u,b\right)\in H^{2}{\left(\Omega \right)}^{d}\times H^{2}{\left(\Omega \right)}^{d},\left(\kappa_{\mu },D_{\mu }\right)\in L^{2}{\left(\Omega \right)}^{d}\times L^{2}{\left(\Omega \right)}^{d},\left(e_{\mu },E_{\mu }\right)\in X_{1\mu }\times X_{2\mu }. \end{array} \end{array}$$

The important lemma followed there, which has been proved in [11,14,37].

**Lemma** **3.** *There exists a constant β>0 such that*

(10) $$\begin{array}{c} \underset{\left(\kappa_{\mu },D_{\mu }\right)\in X_{h}}{\sup}\frac{b(\left(\kappa_{\mu },D_{\mu }\right),p_{\mu })}{∥\left(\kappa_{\mu },D_{\mu }\right){∥}_{1}}\geq \beta {∥p_{\mu }∥}_{0},\;\forall p_{\mu }\in M_{h}. \end{array}$$

Combining with the above conclusions, we can obtain the existence and uniqueness results of (6) from [18].

**Theorem** **3.** Suppose that $\frac{\sqrt{2}\max\left\{N_{1},N_{2}\right\}{∥F∥}_{*}}{{\left(\min\left\{R_{e}^{-1},\lambda_{1}R_{m}^{-1}S_{c}\right\}\right)}^{2}}<1$, (6) is well-posed and its unique solution satisfies

(11) $$\begin{array}{c} ∥(u_{\mu },b_{\mu }){∥}_{1}\leq \frac{{∥F∥}_{*}}{\min\left\{R_{e}^{-1},\lambda_{1}R_{m}^{-1}S_{c}\right\}}. \end{array}$$

For the convenience of the interval, it is expected to be easier to estimate the error of the solutions $\left(\left(u_{\mu },b_{\mu }\right),p_{\mu }\right)\in X_{1\mu }\times X_{2\mu }\times M_{\mu }$. Then, the projection operators $\left(\left(S_{\mu },Q_{\mu }\right),P_{\mu }\right)$ are given as follows: $H_{0}^{1}\left(\Omega \right)\times H_{n}^{1}\left(\Omega \right)\times L_{0}^{2}\left(\Omega \right)⟶X_{1\mu }\times X_{2\mu }\times M_{\mu }$ through

(12) $$\begin{array}{c} \begin{array}{c} A_{0}\left(\left(u-S_{\mu }\left(u,p\right),b-Q_{\mu }\left(b\right)\right),\left(\kappa_{\mu },D_{\mu }\right)\right)-b\left(\left(\kappa_{\mu },D_{\mu }\right),p-P_{\mu }\left(u,p\right)\right)\\ +b\left(\left(u-S_{\mu }\left(u,p\right),b-Q_{\mu }\left(b\right)\right),r_{\mu }\right)=0,\;\forall \left(\left(\kappa_{\mu },D_{\mu }\right),r_{\mu }\right)\in X_{1\mu }\times X_{2\mu }\times M_{\mu }. \end{array} \end{array}$$

As the finite element spaces $X_{1\mu }$, $X_{2\mu }$ and $M_{\mu }$ satisfy property (5), the above projection operators satisfy the following conclusion [16,17]:

**Lemma** **4.** *For all $\left(\kappa ,D\right)\in \left(H_{0}^{1}\left(\Omega \right)\cap H^{2}\left(\Omega \right)\right)\times \left(H_{n}^{1}\left(\Omega \right)\cap H^{2}\left(\Omega \right)\right)$, $r\in \left(L_{0}^{2}\left(\Omega \right)\cap H^{1}\left(\Omega \right)\right)$, the projection operators $\left(\left(S_{\mu },Q_{\mu }\right),P_{\mu }\right)$ satisfy*

(13) $$\begin{array}{c} \begin{array}{c} ∥\left(\kappa -S_{\mu }\left(\kappa ,r\right),D-Q_{\mu }\left(D\right)\right){∥}_{1}+∥(r-P_{\mu }{\left(\kappa ,r\right)∥}_{0}\leq {c\mu (∥\kappa ∥}_{2}+{∥D∥}_{2}+{∥r∥}_{1}). \end{array} \end{array}$$

Next, we draw two important conclusions from this section. One is the error estimate of $H^{1}$-norm of finite element solution without proof, which has been given in [19]. Another important result is the error estimate of $L^{2}$-norm of finite element solution obtained by duality theory [38], which has been proved and can be found in [19].

**Theorem** **4.** Assume that $\frac{\sqrt{2}\max\left\{N_{1},N_{2}\right\}{∥F∥}_{*}}{{\left(\min\left\{R_{e}^{-1},\lambda_{1}R_{m}^{-1}S_{c}\right\}\right)}^{2}}<1$. Let $u\in (H_{0}^{1}\left(\Omega \right)\cap H^{2}\left(\Omega \right))$, $b\in (H_{n}^{1}\left(\Omega \right)\cap H^{2}\left(\Omega \right))$, $p\in (L_{0}^{2}\left(\Omega \right)\cap H^{1}\left(\Omega \right))$ and $\left(u_{\mu },b_{\mu }\right)\in X_{1\mu }\times X_{2\mu }=X_{\mu },p_{\mu }\in M_{\mu }$ be the solutions of (2) and (6), respectively. Then, $\left(u-u_{\mu },b-b_{\mu }\right)$ and $p-p_{\mu }$ satisfy the bound

$$\begin{array}{c} \begin{array}{cc} & {∥\left(u-u_{\mu },b-b_{\mu }\right)∥}_{1}+{∥p-p_{\mu }∥}_{0}\leq C\mu \left({∥u∥}_{2}+{∥b∥}_{2}+{∥p∥}_{1}\right),\\ & {∥\left(u-u_{\mu },b-b_{\mu }\right)∥}_{0}\leq C\mu^{2}\left({∥u∥}_{2}+{∥b∥}_{2}+{∥p∥}_{1}\right). \end{array} \end{array}$$

## 4. Iterative Methods

Recently, for the MHD problem, three iteration methods under different uniqueness conditions are presented in [10] and three iteration methods based on nonconforming FEM are designed in [31] on a Lipschitz domain. Therefore, we further give the important conclusion of this section, three iterative methods on convex region and their error estimates. Three iterative methods appear as follows:

Given $\left(u_{\mu }^{n-1},b_{\mu }^{n-1}\right)\in X_{1\mu }\times X_{2\mu },$ solve $\left(u_{\mu }^{n},b_{\mu }^{n}\right)\in X_{1\mu }\times X_{2\mu },p_{\mu }^{n}\in M_{\mu }$ from

Method 1: the Stokes iteration method.

(14) $$\begin{array}{c} \begin{array}{cc} & A_{0}\left(\left(u_{\mu }^{n},b_{\mu }^{n}\right),\left(\kappa ,D\right)\right)+A_{1}\left(\left(u_{\mu }^{n-1},b_{\mu }^{n-1}\right),\left(u_{\mu }^{n-1},b_{\mu }^{n-1}\right),\left(\kappa ,D\right)\right)-b\left(\left(\kappa ,D\right),p_{\mu }^{n}\right)\\ + & b\left(\left(u_{\mu }^{n},b_{\mu }^{n}\right),r\right)=<F,\left(\kappa ,D\right)>. \end{array} \end{array}$$

Method 2: the Newton iteration method.

(15) $$\begin{array}{c} \begin{array}{cc} & A_{0}\left(\left(u_{\mu }^{n},b_{\mu }^{n}\right),\left(\kappa ,D\right)\right)+A_{1}\left(\left(u_{\mu }^{n-1},b_{\mu }^{n-1}\right),\left(u_{\mu }^{n},b_{\mu }^{n}\right),\left(\kappa ,D\right)\right)\\ + & A_{1}\left(\left(u_{\mu }^{n},b_{\mu }^{n}\right),\left(u_{\mu }^{n-1},b_{\mu }^{n-1}\right),\left(\kappa ,D\right)\right)-b\left(\left(\kappa ,D\right),p_{\mu }^{n}\right)+b\left(\left(u_{\mu }^{n},b_{\mu }^{n}\right),r\right)\\ = & A_{1}\left(\left(u_{\mu }^{n-1},b_{\mu }^{n-1}\right),\left(u_{\mu }^{n-1},b_{\mu }^{n-1}\right),\left(\kappa ,D\right)\right)+<F,\left(\kappa ,D\right)>. \end{array} \end{array}$$

Method 3: the Oseen iteration method.

(16) $$\begin{array}{c} \begin{array}{cc} & A_{0}\left(\left(u_{\mu }^{n},b_{\mu }^{n}\right),\left(\kappa ,D\right)\right)+A_{1}\left(\left(u_{\mu }^{n-1},b_{\mu }^{n-1}\right),\left(u_{\mu }^{n},b_{\mu }^{n}\right),\left(\kappa ,D\right)\right)-b\left(\left(\kappa ,D\right),p_{\mu }^{n}\right)\\ + & b\left(\left(u_{\mu }^{n},b_{\mu }^{n}\right),r\right)=<F,\left(\kappa ,D\right)>. \end{array} \end{array}$$

The initial value $\left(\left(u_{\mu }^{0},b_{\mu }^{0}\right),p_{\mu }^{0}\right)$ is obtained from

(17) $$\begin{array}{c} \begin{array}{c} A_{0}\left(\left(u_{\mu }^{0},b_{\mu }^{0}\right),\left(\kappa ,D\right)\right)-b\left(\left(\kappa ,D\right),p_{\mu }^{0}\right)+b\left(\left(u_{\mu }^{0},b_{\mu }^{0}\right),r\right)=<F,\left(\kappa ,D\right)>. \end{array} \end{array}$$

for all $\left(\kappa_{\mu },D_{\mu }\right)\in X_{1\mu }\times X_{2\mu },r_{\mu }\in M_{\mu }$.

Next, we further find the stability of these iterative methods, which can be proved by using similar methods in [10,31].

**Theorem** **5.** Suppose that $0<\sigma :=\frac{\sqrt{2}\max\left\{N_{1},N_{2}\right\}{∥F∥}_{*}}{{\left(\min\left\{R_{e}^{-1},\lambda_{1}R_{m}^{-1}S_{c}\right\}\right)}^{2}}<\frac{2}{5}$. Then, $\left(u_{\mu }^{n},b_{\mu }^{n}\right)$ defined by iterative method 1 satisfies

(18) $$\begin{array}{c} {∥\left(u_{\mu }^{n},b_{\mu }^{n}\right)∥}_{1}\leq \frac{6}{5}\frac{{∥F∥}_{*}}{\min\left\{R_{e}^{-1},\lambda_{1}R_{m}^{-1}S_{c}\right\}}, \end{array}$$

*and $\left(u_{\mu }-u_{\mu }^{n},b_{\mu }-b_{\mu }^{n}\right)$ and $p_{\mu }-p_{\mu }^{n}$ have the following bounds:*

(19) $$\begin{array}{cc} & {∥\left(u_{\mu }-u_{\mu }^{n},b_{\mu }-b_{\mu }^{n}\right)∥}_{h}\leq {\left(\frac{11}{5}\sigma \right)}^{n}\frac{2}{5}\frac{{∥F∥}_{*}}{\min\left\{R_{e}^{-1},\lambda_{1}R_{m}^{-1}S_{c}\right\}}, \end{array}$$

(20) $$\begin{array}{cc} & {∥p_{\mu }-p_{\mu }^{n}∥}_{0}\leq C{\left(\frac{11}{5}\sigma \right)}^{n}\frac{2}{5}\frac{{∥F∥}_{*}}{\min\left\{R_{e}^{-1},\lambda_{1}R_{m}^{-1}S_{c}\right\}}, \end{array}$$

*for all n≥0.*

**Theorem** **6.** *Suppose that $0<\sigma <\frac{5}{11}$. Then, $\left(u_{\mu }^{n},b_{\mu }^{n}\right)$ defined by iterative method 2 satisfies*

(21) $$\begin{array}{c} {∥\left(u_{\mu }^{n},b_{\mu }^{n}\right)∥}_{1}\leq \frac{4}{3}\frac{{∥F∥}_{*}}{\min\left\{R_{e}^{-1},\lambda_{1}R_{m}^{-1}S_{c}\right\}}, \end{array}$$

*and $\left(u_{\mu }-u_{\mu }^{n},b_{\mu }-b_{\mu }^{n}\right)$ and $p_{\mu }-p_{\mu }^{n}$ have the following bounds:*

(22) $$\begin{array}{cc} & {∥\left(u_{\mu }-u_{\mu }^{n},b_{\mu }-b_{\mu }^{n}\right)∥}_{h}\leq {\left(\frac{15}{13}\sigma \right)}^{2^{n}-1}\frac{5}{11}\frac{{∥F∥}_{*}}{\min\left\{R_{e}^{-1},\lambda_{1}R_{m}^{-1}S_{c}\right\}}, \end{array}$$

(23) $$\begin{array}{cc} & {∥p_{\mu }-p_{\mu }^{n}∥}_{0}\leq C{\left(\frac{15}{13}\sigma \right)}^{2^{n}-1}\frac{5}{11}\frac{{∥F∥}_{*}}{\min\left\{R_{e}^{-1},\lambda_{1}R_{m}^{-1}S_{c}\right\}}, \end{array}$$

*for all n≥0.*

**Theorem** **7.** *Suppose that 0<σ<1. Then, $\left(u_{\mu }^{n},b_{\mu }^{n}\right)$ defined by the iterative method 3 satisfies*

(24) $$\begin{array}{c} {∥\left(u_{\mu }^{n},b_{\mu }^{n}\right)∥}_{1}\leq \frac{{∥F∥}_{*}}{\min\left\{R_{e}^{-1},\lambda_{1}R_{m}^{-1}S_{c}\right\}}, \end{array}$$

*and $\left(u_{\mu }-u_{\mu }^{n},b_{\mu }-b_{\mu }^{n}\right)$ and $p_{\mu }-p_{\mu }^{n}$ have the following bounds:*

(25) $$\begin{array}{cc} & {∥\left(u_{\mu }-u_{\mu }^{n},b_{\mu }-b_{\mu }^{n}\right)∥}_{1}\leq \sigma^{n}\frac{{∥F∥}_{*}}{\min\left\{R_{e}^{-1},\lambda_{1}R_{m}^{-1}S_{c}\right\}}, \end{array}$$

(26) $$\begin{array}{cc} & {∥p_{\mu }-p_{\mu }^{n}∥}_{0}\leq C\sigma^{n}\frac{{∥F∥}_{*}}{\min\left\{R_{e}^{-1},\lambda_{1}R_{m}^{-1}S_{c}\right\}}, \end{array}$$

*for all n≥0.*

According to Theorems 4–7, we draw the important conclusions of this section as follows.

**Theorem** **8.** If $0<\sigma :=\frac{\sqrt{2}\max\left\{N_{1},N_{2}\right\}{∥F∥}_{*}}{{\left(\min\left\{R_{e}^{-1},\lambda_{1}R_{m}^{-1}S_{c}\right\}\right)}^{2}}<\frac{2}{5}$, $\left(u-u_{\mu }^{n},b-b_{\mu }^{n}\right)$ and $p-p_{\mu }^{n}$ satisfy

(27) $$\begin{array}{cc} & {∥\left(u-u_{\mu }^{n},b-b_{\mu }^{n}\right)∥}_{1}\leq C\mu \left({∥u∥}_{2}+{∥b∥}_{2}+{∥p∥}_{1}\right)+{\left(\frac{11}{5}\sigma \right)}^{n}\frac{2}{5}\frac{{∥F∥}_{*}}{\min\left\{R_{e}^{-1},\lambda_{1}R_{m}^{-1}S_{c}\right\}}, \end{array}$$

(28) $$\begin{array}{cc} & {∥p-p_{\mu }^{n}∥}_{0}\leq C\mu \left({∥u∥}_{2}+{∥b∥}_{2}+{∥p∥}_{1}\right)+C{\left(\frac{11}{5}\sigma \right)}^{n}\frac{2}{5}\frac{{∥F∥}_{*}}{\min\left\{R_{e}^{-1},\lambda_{1}R_{m}^{-1}S_{c}\right\}}, \end{array}$$

*for all n≥0; If $0<\sigma <\frac{5}{11}$, $\left(u-u_{\mu }^{n},b-b_{\mu }^{n}\right)$ and $p-p_{\mu }^{n}$ satisfy*

(29) $$\begin{array}{cc} & {∥\left(u-u_{\mu }^{n},b-b_{\mu }^{n}\right)∥}_{1}\leq C\mu \left({∥u∥}_{2}+{∥b∥}_{2}+{∥p∥}_{1}\right)+{\left(\frac{15}{13}\sigma \right)}^{2^{n}-1}\frac{5}{11}\frac{{∥F∥}_{*}}{\min\left\{R_{e}^{-1},\lambda_{1}R_{m}^{-1}S_{c}\right\}}, \end{array}$$

(30) $$\begin{array}{cc} & {∥p-p_{\mu }^{n}∥}_{0}\leq C\mu \left({∥u∥}_{2}+{∥b∥}_{2}+{∥p∥}_{1}\right)+C{\left(\frac{15}{13}\sigma \right)}^{2^{n}-1}\frac{5}{11}\frac{{∥F∥}_{*}}{\min\left\{R_{e}^{-1},\lambda_{1}R_{m}^{-1}S_{c}\right\}}, \end{array}$$

*for all n≥0; If 0<σ<1, $\left(u-u_{\mu }^{n},b-b_{\mu }^{n}\right)$ and $p-p_{\mu }^{n}$ satisfy*

(31) $$\begin{array}{cc} & {∥\left(u-u_{\mu }^{n},b-b_{\mu }^{n}\right)∥}_{1}\leq C\mu \left({∥u∥}_{2}+{∥b∥}_{2}+{∥p∥}_{1}\right)+\sigma^{n}\frac{{∥F∥}_{*}}{\min\left\{R_{e}^{-1},\lambda_{1}R_{m}^{-1}S_{c}\right\}}, \end{array}$$

(32) $$\begin{array}{cc} & {∥p-p_{\mu }^{n}∥}_{0}\leq C\mu \left({∥u∥}_{2}+{∥b∥}_{2}+{∥p∥}_{1}\right)+C\sigma^{n}\frac{{∥F∥}_{*}}{\min\left\{R_{e}^{-1},\lambda_{1}R_{m}^{-1}S_{c}\right\}}, \end{array}$$

*for all n≥0.*

For further clarification about all the methods, we describe them as follows.

**Remark** **2.** *From the above discussion, we can see clearly that method 1 is the simplest. In the condition $0<\sigma <\frac{5}{11}$, method 2 is stable and has exponential convergence rate for n, which is faster than methods 1 and 3. In addition, if 0<σ<1, method 3 is stable and convergent and has the widest application scope.*

## 5. Two-Level Iterative Methods

Some two-level methods for solving the MHD problem with different Reynolds numbers are proposed in [30]. In this section, based on nonconforming FEM, to further study more effective two-level methods, several two-level methods are proposed in different conditions as follows.

### 5.1. Two-Level Iterative Method with $0<\sigma <\frac{2}{5}$

If $0<\sigma <\frac{2}{5}$, we know that three iterative methods are all stable and convergent. We firstly propose nine two-level methods to derive the iterative solution $\left(\left(u_{H}^{m},b_{H}^{m}\right),p_{H}^{m}\right)$ using methods 1–3 on coarse mesh $T_{H}$, and then to solve the correction solution $\left(\left(u_{mh},b_{mh}\right),p_{mh}\right)$ using corrections 1–3 on fine mesh $T_{h}$. Here, *H* and *h* are set as positive numbers tending to zero (0<h≤H). All of the proposed methods can be shown below:

**Step 1**. Solve the MHD equations on the coarse grid, $\left(\left(u_{H}^{m},b_{H}^{m}\right),p_{H}^{m}\right)\in X_{H}\times M_{H}$ provided by methods 1–3, respectively.

**Step 2**. One step correction on the fine grid: find $\left(\left(u_{mh},b_{mh}\right),p_{mh}\right)\in X_{h}\times M_{h}$ satisfy:

Correction 1: Stokes correction.

(33) $$\begin{array}{c} \begin{array}{cc} & A_{0}\left(\left(u_{mh},b_{mh}\right),\left(\kappa ,D\right)\right)-b\left(\left(\kappa ,D\right),p_{mh}\right)+b\left(\left(u_{mh},b_{mh}\right),r\right)\\ = & -A_{1}\left(\left(u_{H}^{m},b_{H}^{m}\right),\left(u_{H}^{m},b_{H}^{m}\right),\left(\kappa ,D\right)\right)+<F,\left(\kappa ,D\right)>. \end{array} \end{array}$$

Correction 2: Newton correction.

(34) $$\begin{array}{c} \begin{array}{cc} & A_{0}\left(\left(u_{mh},b_{mh}\right),\left(\kappa ,D\right)\right)-b\left(\left(\kappa ,D\right),p_{mh}\right)+b\left(\left(u_{mh},b_{mh}\right),r\right)\\ + & A_{1}\left(\left(u_{H}^{m},b_{H}^{m}\right),\left(u_{mh},b_{mh}\right),\left(\kappa ,D\right)\right)+A_{1}\left(\left(u_{mh},b_{mh}\right),\left(u_{H}^{m},b_{H}^{m}\right),\left(\kappa ,D\right)\right)\\ = & -A_{1}\left(\left(u_{H}^{m},b_{H}^{m}\right),\left(u_{H}^{m},b_{H}^{m}\right),\left(\kappa ,D\right)\right)+<F,\left(\kappa ,D\right)>. \end{array} \end{array}$$

Correction 3: Oseen correction.

(35) $$\begin{array}{c} \begin{array}{cc} & A_{0}\left(\left(u_{mh},b_{mh}\right),\left(\kappa ,D\right)\right)-b\left(\left(\kappa ,D\right),p_{mh}\right)+b\left(\left(u_{mh},b_{mh}\right),r\right)\\ = & -A_{1}\left(\left(u_{H}^{m},b_{H}^{m}\right),\left(u_{mh},b_{mh}\right),\left(\kappa ,D\right)\right)+<F,\left(\kappa ,D\right)>. \end{array} \end{array}$$

for all $\left(\left(\kappa ,D\right),r\right)\in X_{h}\times M_{h}$.

In what follows, we set out to give the following results for the above two-level iterative methods by introducing projection operators.

**Theorem** **9.** *Under the conditions of Theorem 4 and $0<\sigma <\frac{2}{5}$, $\left(\left(u_{H}^{m},b_{H}^{m}\right),p_{H}^{m}\right)\in X_{H}\times M_{H}$ calculated by methods 1–3, $(\left(u_{mh},b_{mh}\right)$, $p_{mh})$ calculated by correction 1. Then, $\left(u-u_{mh},b-b_{mh}\right)$ and $p-p_{mh}$ satisfy:*

$$\begin{array}{c} \begin{array}{cc} {∥\left(u-u_{mh},b-b_{mh}\right)∥}_{1}\leq & C\left(h+H^{2}\right){(∥u∥}_{2}+{∥p∥}_{1}+{∥b∥}_{2})+C∥\left(u_{H}-u_{H}^{m},b_{H}-b_{H}^{m}\right){∥}_{1},\\ {∥p-p_{mh}∥}_{0}\leq C(h+ & H^{2}{)(∥u∥}_{2}+{∥p∥}_{1}+{∥b∥}_{2})+C∥\left(u_{H}-u_{H}^{m},b_{H}-b_{H}^{m}\right){∥}_{1}, \end{array} \end{array}$$

*for all m≥0.*

**Proof.**  For $\left(\kappa ,D\right)\in X_{h}$, $r\in M_{h}$, we have

(36) $$\begin{array}{c} \begin{array}{cc} & A_{0}\left(\left(u,b\right),\left(\kappa ,D\right)\right)+A_{1}\left(\left(u,b\right),\left(u,b\right),\left(\kappa ,D\right)\right)-b\left(\left(\kappa ,D\right),p\right)+b\left(\left(u,b\right),r\right)\\ - & <F,\left(\kappa ,D\right)>=E\left(\left(\kappa ,D\right)\right), \end{array} \end{array}$$

where

$$\begin{array}{c} E\left(\left(\kappa ,D\right)\right)=\sum_{K\in T_{h}}\int_{\partial K}\left[R_{e}^{-1}\frac{\partial u}{\partial n}\kappa -p\kappa \cdot n-\frac{1}{2}\left(u\cdot n\right)\left(u\cdot \kappa \right)\right]ds. \end{array}$$

Then, we can derive the error equation by subtract (33) from (36),

(37) $$\begin{array}{c} \begin{array}{cc} & A_{0}\left(\left(u-u_{mh},b-b_{mh}\right),\left(\kappa ,D\right)\right)+A_{1}\left(\left(u,b\right),\left(u-u_{H}^{m},b-b_{H}^{m}\right),\left(\kappa ,D\right)\right)\\ + & A_{1}\left(\left(u-u_{H}^{m},b-b_{H}^{m}\right),\left(u,b\right),\left(\kappa ,D\right)\right)-b\left(\left(v,\Upsilon \right),p-p_{mh}\right)\\ + & b(\left(u-u_{mh},b-b_{mh}\right),r)\\ = & A_{1}\left(\left(u-u_{H}^{m},b-b_{H}^{m}\right),\left(u-u_{H}^{m},b-b_{H}^{m}\right),\left(\kappa ,D\right)\right)+E\left(\left(\kappa ,D\right)\right). \end{array} \end{array}$$

Subtracting (37) from (12), we obtain

(38) $$\begin{array}{c} \begin{array}{cc} & A_{0}\left(\left(S_{h}\left(u,p\right)-u_{mh},Q_{h}\left(b\right)-b_{mh}\right),\left(\kappa ,D\right)\right)+A_{1}\left(\left(u-u_{H},b-b_{H}\right),\left(u,b\right),\left(\kappa ,D\right)\right)\\ + & A_{1}\left(\left(u_{H}-u_{H}^{m},b_{H}-b_{H}^{m}\right),\left(u,b\right),\left(\kappa ,D\right)\right)+A_{1}\left(\left(u,b\right),\left(u-u_{H},b-b_{H}\right),\left(\kappa ,D\right)\right)\\ + & A_{1}\left(\left(u,b\right),\left(u_{H}-u_{H}^{m},b_{H}-b_{H}^{m}\right),\left(\kappa ,D\right)\right)-b\left(\left(\kappa ,D\right),P_{h}\left(u,p\right)-p_{mh}\right)\\ + & b(\left(S_{h}\left(u,p\right)-u_{mh},Q_{h}\left(b\right)-b_{mh}\right),r)\\ = & A_{1}\left(\left(u-u_{H}^{m},b-b_{H}^{m}\right),\left(u-u_{H}^{m},b-b_{H}^{m}\right),\left(\kappa ,D\right)\right)+E\left(\left(\kappa ,D\right)\right). \end{array} \end{array}$$

Setting $\left(\kappa ,D\right)=(S_{h}\left(u,p\right)-u_{mh},Q_{h}\left(b\right)-b_{mh})$, $r=P_{h}\left(u,p\right)-p_{mh}$ in (38), and applying the property (13), we deduce

(39) $$\begin{array}{c} \begin{array}{cc} & ∥\left(S_{h}\left(u,p\right)-u_{mh},Q_{h}\left(b\right)-b_{mh}\right){∥}_{1}\\ \leq & \frac{2C}{\min\left\{R_{e}^{-1},\lambda_{1}R_{m}^{-1}S_{c}\right\}}{∥\left(u,b\right)∥}_{2}{∥\left(u-u_{H},b-b_{H}\right)∥}_{0}\\ + & \frac{2\sqrt{2}\max\left\{N_{1},N_{2}\right\}}{\min\left\{R_{e}^{-1},\lambda_{1}R_{m}^{-1}S_{c}\right\}}{∥\left(u,b\right)∥}_{1}{∥\left(u_{H}-u_{H}^{m},b_{H}-b_{H}^{m}\right)∥}_{1}\\ + & \frac{\sqrt{2}\max\left\{N_{1},N_{2}\right\}}{\min\left\{R_{e}^{-1},\lambda_{1}R_{m}^{-1}S_{c}\right\}}{∥\left(u-u_{H}^{m},b-b_{H}^{m}\right)∥}_{1}^{2}\\ + & \frac{ch}{\min\left\{R_{e}^{-1},\lambda_{1}R_{m}^{-1}S_{c}\right\}}{(∥u∥}_{2}+{∥p∥}_{1}). \end{array} \end{array}$$

Then, using (13), (39) and the triangle inequality, we can hit bottom

$$\begin{array}{c} \begin{array}{cc} & ∥(u-u_{mh},b-b_{mh}{∥}_{1}\\ \leq & ∥\left(u-S_{h}\left(u,p\right),b-Q_{h}\left(b\right)\right){∥}_{1}+{∥\left(S_{h}\left(u,p\right)-u_{mh},Q_{h}\left(b\right)-b_{mh}\right)∥}_{1}\\ \leq & C\left(h+H^{2}\right){(∥u∥}_{2}+{∥p∥}_{1}+{∥b∥}_{2})+C∥\left(u_{H}-u_{H}^{m},b_{H}-b_{H}^{m}\right){∥}_{1}. \end{array} \end{array}$$

Next, using the property (7) and (9) in (38) yields

(40) $$\begin{array}{c} \begin{array}{cc} {∥P_{h}\left(u,p\right)-p_{mh}∥}_{0}\leq & \frac{\max\{R_{e}^{-1},\left(2+d\right)S_{c}R_{m}^{-1}\}}{\beta }{∥\left(S_{h}\left(u,p\right)-u_{mh},Q_{h}\left(b\right)-b_{mh}\right)∥}_{1}\\ + & \frac{2C}{\beta }{∥\left(u-u_{H}^{m},b-b_{H}^{m}\right)∥}_{0}{∥\left(u,b\right)∥}_{2}+\frac{ch}{\beta }{(∥u∥}_{2}+{∥p∥}_{1})\\ + & \frac{2\sqrt{2}\max\left\{N_{1},N_{2}\right\}}{\beta }{∥\left(u-u_{H}^{m},b-b_{H}^{m}\right)∥}_{1}{∥\left(u,b\right)∥}_{1}\\ + & \frac{\sqrt{2}\max\left\{N_{1},N_{2}\right\}}{\beta }{∥\left(u-u_{H}^{m},b-b_{H}^{m}\right)∥}_{1}^{2}. \end{array} \end{array}$$

Finally, we can obtain the conclusion by applying (13), (40) and the triangle inequality,

$$\begin{array}{c} \begin{array}{cc} {∥p-p_{mh}∥}_{0}\leq & {∥p-P_{h}\left(u,p\right)∥}_{0}+{∥P_{h}\left(u,p\right)-p_{mh}∥}_{0}\\ & \leq C\left(h+H^{2}\right){(∥u∥}_{2}+{∥p∥}_{1}+{∥b∥}_{2})+C∥\left(u_{H}-u_{H}^{m},b_{H}-b_{H}^{m}\right){∥}_{1}. \end{array} \end{array}$$

Thus, the proof is done. □

**Theorem** **10.** *Under the conditions of Theorem 4 and $0<\sigma <\frac{2}{5}$, $\left(\left(u_{H}^{m},b_{H}^{m}\right),p_{H}^{m}\right)\in X_{H}\times M_{H}$ calculated by methods 1–3, and $(\left(u_{mh},b_{mh}\right)$, $p_{mh})$ was calculated by correction 2. Then, $\left(u-u_{mh},b-b_{mh}\right)$ and $p-p_{mh}$ satisfy:*

$$\begin{array}{cc} & {∥\left(u-u_{mh},b-b_{mh}\right)∥}_{1}\leq C\left(h+H^{2}\right){(∥u∥}_{2}+{∥p∥}_{1}+{∥b∥}_{2})+C∥\left(u_{H}-u_{H}^{m},b_{H}-b_{H}^{m}\right){∥}_{1}^{2},\\ & {∥p-p_{mh}∥}_{0}\leq C\left(h+H^{2}\right){(∥u∥}_{2}+{∥p∥}_{1}+{∥b∥}_{2})+C∥\left(u_{H}-u_{H}^{m},b_{H}-b_{H}^{m}\right){∥}_{1}^{2}, \end{array}$$

*for all m≥0.*

**Proof.**  Firstly, by subtracting (34) from (36), we can obtain

(41) $$\begin{array}{c} \begin{array}{cc} & A_{0}\left(\left(u-u_{mh},b-b_{mh}\right),\left(\kappa ,D\right)\right)-b\left(\left(\kappa ,D\right),p-p_{mh}\right)+b\left(\left(u-u_{mh},b-b_{mh}\right),r\right)\\ = & A_{1}\left(\left(u_{H}^{m},b_{H}^{m}\right),\left(u-u_{mh},b-b_{mh}\right),\left(\kappa ,D\right)\right)\\ + & A_{1}\left(\left(u-u_{H}^{m},b-b_{H}^{m}\right),\left(u-u_{H}^{m},b-b_{H}^{m}\right),\left(\kappa ,D\right)\right)\\ + & A_{1}\left(\left(u-u_{mh},b-b_{mh}\right),\left(u_{H}^{m},b_{H}^{m}\right),\left(\kappa ,D\right)\right)+E\left(\left(\kappa ,D\right)\right), \end{array} \end{array}$$

Subtracting (41) from (12), we derive

(42) $$\begin{array}{c} \begin{array}{cc} & A_{0}\left(\left(S_{h}\left(u,p\right)-u_{mh},Q_{h}\left(b\right)-b_{mh}\right),\left(\kappa ,D\right)\right)-b\left(\left(\kappa ,D\right),P_{h}\left(u,p\right)-p_{mh}\right)\\ + & b(\left(S_{h}\left(u,p\right)-u_{mh},Q_{h}\left(b\right)-b_{mh}\right),r)\\ = & A_{1}\left(\left(u_{H}^{m},b_{H}^{m}\right),\left(u-S_{h}\left(u,p\right),b-Q_{h}\left(b\right)\right),\left(\kappa ,D\right)\right)\\ + & A_{1}\left(\left(u_{H}^{m},b_{H}^{m}\right),\left(S_{h}\left(u,p\right)-u_{mh},Q_{h}\left(b\right)-b_{mh}\right),\left(\kappa ,D\right)\right)\\ + & A_{1}(\left(u-S_{h}\left(u,p\right),b-Q_{h}\left(b\right),\left(u_{H}^{m},b_{H}^{m}\right),\left(\kappa ,D\right)\right)\\ + & A_{1}(\left(S_{h}\left(u,p\right)-u_{mh},Q_{h}\left(b-b_{mh}\right),\left(u_{H}^{m},b_{H}^{m}\right),\left(\kappa ,D\right)\right)\\ + & A_{1}\left(\left(u-u_{H}^{m},b-b_{H}^{m}\right),\left(u-u_{H}^{m},b-b_{H}^{m}\right),\left(\kappa ,D\right)\right)+E\left(\left(\kappa ,D\right)\right). \end{array} \end{array}$$

Letting $\left(\kappa ,D\right)=(S_{h}\left(u,p\right)-u_{mh},Q_{h}\left(b\right)-b_{mh})$, $r=P_{h}\left(u,p\right)-p_{mh}$ in (42), and making the use of (13), we obtain

(43) $$\begin{array}{c} \begin{array}{cc} & \left[\min\left\{R_{e}^{-1},\lambda_{1}R_{m}^{-1}S_{c}\right\}-\sqrt{2}\max\left\{N_{1},N_{2}\right\}∥\left(u_{H}^{m},b_{H}^{m}\right){∥}_{1}\right]\\ \times & ∥\left(S_{h}\left(u,p\right)-u_{mh},Q_{h}\left(b\right)-b_{mh}\right){∥}_{1}\\ \leq & 2\sqrt{2}\max\left\{N_{1},N_{2}\right\}∥\left(u_{H}^{m},b_{H}^{m}\right){∥}_{1}{∥\left(u-S_{h}\left(u,p\right),b-Q_{h}\left(b\right)\right)∥}_{1}\\ + & \sqrt{2}\max\left\{N_{1},N_{2}\right\}∥\left(u-u_{H}^{m},b-b_{H}^{m}\right){∥}_{1}^{2}+{ch(∥u∥}_{2}+{∥p∥}_{1}). \end{array} \end{array}$$

Then, using (13), (43) and the triangle inequality, we can come to the conclusion that

$$\begin{array}{c} \begin{array}{cc} & ∥(u-u_{mh},b-b_{mh}{∥}_{1}\\ \leq & ∥\left(u-S_{h}\left(u,p\right),b-Q_{h}\left(b\right)\right){∥}_{1}+{∥\left(S_{h}\left(u,p\right)-u_{mh},Q_{h}\left(b\right)-b_{mh}\right)∥}_{1}\\ \leq & C\left(h+H^{2}\right){(∥u∥}_{2}+{∥p∥}_{1}+{∥b∥}_{2})+C∥\left(u_{H}-u_{H}^{m},b_{H}-b_{H}^{m}\right){∥}_{1}^{2}. \end{array} \end{array}$$

Next, we can deduce the conclusion by using properties (7) and (9) in (42),

(44) $$\begin{array}{c} \begin{array}{cc} & {∥P_{h}\left(u,p\right)-p_{mh}∥}_{0}\\ \leq & \frac{\max\{R_{e}^{-1},\left(2+d\right)S_{c}R_{m}^{-1}\}}{\beta }{∥\left(S_{h}\left(u,p\right)-u_{mh},Q_{h}\left(b\right)-b_{mh}\right)∥}_{1}\\ + & \frac{2\sqrt{2}\max\left\{N_{1},N_{2}\right\}}{\beta }{∥\left(u_{H}^{m},b_{H}^{m}\right)∥}_{1}{∥\left(u-S_{h}\left(u,p\right),b-Q_{h}\left(b\right)\right)∥}_{1}\\ + & \frac{2\sqrt{2}\max\left\{N_{1},N_{2}\right\}}{\beta }{∥\left(u_{H}^{m},b_{H}^{m}\right)∥}_{1}{∥\left(S_{h}\left(u,p\right)-u_{mh},Q_{h}\left(b\right)-b_{mh})\right)∥}_{1}\\ + & \frac{\sqrt{2}\max\left\{N_{1},N_{2}\right\}}{\beta }{∥\left(u-u_{H}^{m},b-b_{H}^{m}\right)∥}_{1}^{2}+\frac{ch}{\beta }{(∥u∥}_{2}+{∥p∥}_{1}). \end{array} \end{array}$$

Finally, we can obtain the conclusion by applying (13), (44) and the triangle inequality,

$$\begin{array}{c} \begin{array}{cc} {∥p-p_{mh}∥}_{0}\leq & {∥p-P_{h}\left(u,p\right)∥}_{0}+{∥P_{h}\left(u,p\right)-p_{mh}∥}_{0}\\ \leq & C\left(h+H^{2}\right){(∥u∥}_{2}+{∥p∥}_{1}+{∥b∥}_{2})+C∥\left(u_{H}-u_{H}^{m},b_{H}-b_{H}^{m}\right){∥}_{1}^{2}. \end{array} \end{array}$$

Thus, the proof is done. □

**Theorem** **11.** *Under the conditions of Theorem 4 and the condition $0<\sigma <\frac{2}{5}$, $\left(\left(u_{H}^{m},b_{H}^{m}\right),p_{H}^{m}\right)\in X_{H}\times M_{H}$ calculated by methods 1–3, $(\left(u_{mh},b_{mh}\right)$, $p_{mh})$ calculated by correction 3. Then, $\left(u-u_{mh},b-b_{mh}\right)$ and $p-p_{mh}$ satisfy:*

$$\begin{array}{cc} & {∥\left(u-u_{mh},b-b_{mh}\right)∥}_{1}\leq C\left(h+H^{2}\right){(∥u∥}_{2}+{∥p∥}_{1}+{∥b∥}_{2})+C∥\left(u_{H}-u_{H}^{m},b_{H}-b_{H}^{m}\right){∥}_{1},\\ & {∥p-p_{mh}∥}_{0}\leq C\left(h+H^{2}\right){(∥u∥}_{2}+{∥p∥}_{1}+{∥b∥}_{2})+C∥\left(u_{H}-u_{H}^{m},b_{H}-b_{H}^{m}\right){∥}_{1}, \end{array}$$

*for all m≥0.*

**Proof.**  Firstly, by subtracting (35) from (36), we can deduce that

(45) $$\begin{array}{c} \begin{array}{cc} & A_{0}\left(\left(u-u_{mh},b-b_{mh}\right),\left(\kappa ,D\right)\right)+A_{1}\left(\left(u_{H}^{m},b_{H}^{m}\right),\left(u-u_{mh},b-b_{mh}\right),\left(\kappa ,D\right)\right)\\ + & A_{1}\left(\left(u-u_{H}^{m},b-b_{H}^{m}\right),\left(u,b\right),\left(\kappa ,D\right)\right)-b\left(\left(\kappa ,D\right),p-p_{mh}\right)+b\left(\left(u-u_{mh},b-b_{mh}\right),r\right)\\ = & E\left(\left(\kappa ,D\right)\right), \end{array} \end{array}$$

Subtracting (45) from (12), we get

(46) $$\begin{array}{c} \begin{array}{cc} & A_{0}\left(\left(S_{h}\left(u,p\right)-u_{mh},Q_{h}\left(b\right)-b_{mh}\right),\left(\kappa ,D\right)\right)-b\left(\left(\kappa ,D\right),P_{h}\left(u,p\right)-p_{mh}\right)\\ + & b(\left(S_{h}\left(u,p\right)-u_{mh},Q_{h}\left(b\right)-b_{mh}\right),r)\\ + & A_{1}\left(\left(u_{H}^{m},b_{H}^{m}\right),\left(u-S_{h}\left(u,p\right),b-Q_{h}\left(b\right)\right),\left(\kappa ,D\right)\right)\\ + & A_{1}\left(\left(u-u_{H},b-b_{H}\right),\left(u,b\right),\left(\kappa ,D\right)\right)\\ + & A_{1}\left(\left(u_{H}^{m},b_{H}^{m}\right),\left(S_{h}\left(u,p\right)-u_{mh},Q_{h}\left(b\right)-b_{mh}\right),\left(\kappa ,D\right)\right)\\ + & A_{1}\left(\left(u_{H}-u_{H}^{m},b_{H}-b_{H}^{m}\right),\left(u,b\right),\left(\kappa ,D\right)\right)=E\left(\left(\kappa ,D\right)\right). \end{array} \end{array}$$

Letting $\left(\kappa ,D\right)=(S_{h}\left(u,p\right)-u_{mh},Q_{h}\left(b\right)-b_{mh})$, $r=P_{h}\left(u,p\right)-p_{mh}$ in (46), and making the use of (13), we can derive

(47) $$\begin{array}{c} \begin{array}{cc} & ∥\left(S_{h}\left(u,p\right)-u_{mh},Q_{h}\left(b\right)-b_{mh}\right){∥}_{1}\\ \leq & \frac{C}{\min\left\{R_{e}^{-1},\lambda_{1}R_{m}^{-1}S_{c}\right\}}∥\left(u-u_{H},b-b_{H}\right){∥}_{0}{∥\left(u,b\right)∥}_{2}\\ + & \frac{\sqrt{2}\max\left\{N_{1},N_{2}\right\}}{\min\left\{R_{e}^{-1},\lambda_{1}R_{m}^{-1}S_{c}\right\}}∥\left(u_{H}-u_{H}^{m},b_{H}-b_{H}^{m}\right){∥}_{1}∥\left(u,b\right){)∥}_{1}\\ + & \frac{\sqrt{2}\max\left\{N_{1},N_{2}\right\}}{\min\left\{R_{e}^{-1},\lambda_{1}R_{m}^{-1}S_{c}\right\}}∥\left(u_{H}^{m},b_{H}^{m}\right){∥}_{1}{∥\left(u-S_{h}\left(u,p\right),b-Q_{h}\left(b\right)\right)∥}_{1}\\ + & \frac{ch}{\min\left\{R_{e}^{-1},\lambda_{1}R_{m}^{-1}S_{c}\right\}}{(∥u∥}_{2}+{∥p∥}_{1}). \end{array} \end{array}$$

Then, using (13), (47) and the triangle inequality, we can come to the conclusion that

$$\begin{array}{c} \begin{array}{cc} & ∥(u-u_{mh},b-b_{mh}{∥}_{1}\\ \leq & ∥\left(u-S_{h}\left(u,p\right),b-Q_{h}\left(b\right)\right){∥}_{1}+{∥\left(S_{h}\left(u,p\right)-u_{mh},Q_{h}\left(b\right)-b_{mh}\right)∥}_{1}\\ \leq & C\left(h+H^{2}\right){(∥u∥}_{2}+{∥p∥}_{1}+{∥b∥}_{2})+C∥\left(u_{H}-u_{H}^{m},b_{H}-b_{H}^{m}\right){∥}_{1}. \end{array} \end{array}$$

Next, by using (7) and (9) in (46), we deduce that

(48) $$\begin{array}{c} \begin{array}{cc} & {∥P_{h}\left(u,p\right)-p_{mh}∥}_{0}\\ \leq & \frac{\max\{R_{e}^{-1},\left(2+d\right)S_{c}R_{m}^{-1}\}}{\beta }{∥\left(S_{h}\left(u,p\right)-u_{mh},Q_{h}\left(b\right)-b_{mh}\right)∥}_{1}\\ + & \frac{C}{\beta }∥\left(u-u_{H},b-b_{H}\right){∥}_{0}{∥\left(u,b\right)∥}_{2}\\ + & \frac{\sqrt{2}\max\left\{N_{1},N_{2}\right\}}{\beta }{∥\left(u_{H}^{m},b_{H}^{m}\right)∥}_{1}{∥\left(u-S_{h}\left(u,p\right),b-Q_{h}\left(b\right)\right)∥}_{1}\\ + & \frac{\sqrt{2}\max\left\{N_{1},N_{2}\right\}}{\beta }{∥\left(u_{H}-u_{H}^{m},b_{H}-b_{H}^{m}\right)∥}_{1}{∥\left(u,b\right)∥}_{1}+\frac{ch}{\beta }{(∥u∥}_{2}+{∥p∥}_{1})\\ + & \frac{\sqrt{2}\max\left\{N_{1},N_{2}\right\}}{\beta }{∥\left(u_{H}^{m},b_{H}^{m}\right)∥}_{1}{∥\left(S_{h}\left(u,p\right)-u_{mh},Q_{h}\left(b\right)-b_{mh})\right)∥}_{1}. \end{array} \end{array}$$

Finally, we can obtain the conclusion by applying (13), (48) and the triangle inequality,

$$\begin{array}{c} \begin{array}{cc} {∥p-p_{mh}∥}_{0}\leq & {∥p-P_{h}\left(u,p\right)∥}_{0}+{∥P_{h}\left(u,p\right)-p_{mh}∥}_{0}\\ \leq & C\left(h+H^{2}\right){(∥u∥}_{2}+{∥p∥}_{1}+{∥b∥}_{2})+C∥\left(u_{H}-u_{H}^{m},b_{H}-b_{H}^{m}\right){∥}_{1}. \end{array} \end{array}$$

Thus, the proof is done. □

### 5.2. Two-Level Iterative Method with $\frac{2}{5}<\sigma <\frac{5}{11}$

If $\frac{2}{5}<\sigma <\frac{5}{11}$, we know that iterative methods 2 and 3 are stable and convergent, while method 1 is not convergent. We propose six two-level iterative methods to solve the iterative solution $\left(\left(u_{H}^{m},b_{H}^{m}\right),p_{H}^{m}\right)$ using methods 2 and 3 on coarse mesh $T_{H}$, and then to solve the correction solution $\left(\left(u_{mh},b_{mh}\right),p_{mh}\right)$ using corrections 1–3 on fine mesh $T_{h}$. All of the two-level methods are shown below:

**Step 1**. Solve the MHD equations on the coarse grid, $\left(\left(u_{H}^{m},b_{H}^{m}\right),p_{H}^{m}\right)\in X_{H}\times M_{H}$ provided by methods 2 and 3, respectively.

**Step 2**. One step correction on the fine grid, $\left(\left(u_{mh},b_{mh}\right),p_{mh}\right)\in X_{h}\times M_{h}$ provided by corrections 1–3, respectively.

Then, we further give the following important results about the estimation of the above two-level methods.

**Theorem** **12.** *Under the conditions of Theorem 4 and the condition $\frac{2}{5}<\sigma <\frac{5}{11}$, $\left(\left(u_{H}^{m},b_{H}^{m}\right),p_{H}^{m}\right)\in X_{H}\times M_{H}$ calculated by methods 2 and 3, $(\left(u_{mh},b_{mh}\right)$, $p_{mh})$ calculated by correction 1. Then, $\left(u-u_{mh},b-b_{mh}\right)$ and $p-p_{mh}$ satisfy:*

$$\begin{array}{c} \begin{array}{cc} {∥\left(u-u_{mh},b-b_{mh}\right)∥}_{1}\leq & C\left(h+H^{2}\right){(∥u∥}_{2}+{∥p∥}_{1}+{∥b∥}_{2})+C∥\left(u_{H}-u_{H}^{m},b_{H}-b_{H}^{m}\right){∥}_{1},\\ {∥p-p_{mh}∥}_{0}\leq C(h+ & H^{2}{)(∥u∥}_{2}+{∥p∥}_{1}+{∥b∥}_{2})+C∥\left(u_{H}-u_{H}^{m},b_{H}-b_{H}^{m}\right){∥}_{1}, \end{array} \end{array}$$

*and $\left(\left(u_{mh},b_{mh}\right),p_{mh}\right)$ calculated by correction 2, $\left(u-u_{mh},b-b_{mh}\right)$ and $p-p_{mh}$ satisfy:*

$$\begin{array}{cc} & {∥\left(u-u_{mh},b-b_{mh}\right)∥}_{1}\leq C\left(h+H^{2}\right){(∥u∥}_{2}+{∥p∥}_{1}+{∥b∥}_{2})+C∥\left(u_{H}-u_{H}^{m},b_{H}-b_{H}^{m}\right){∥}_{1}^{2},\\ & {∥p-p_{mh}∥}_{0}\leq C\left(h+H^{2}\right){(∥u∥}_{2}+{∥p∥}_{1}+{∥b∥}_{2})+C∥\left(u_{H}-u_{H}^{m},b_{H}-b_{H}^{m}\right){∥}_{1}^{2}, \end{array}$$

*and $\left(\left(u_{mh},b_{mh}\right),p_{mh}\right)$ calculated by correction 3, $\left(u-u_{mh},b-b_{mh}\right)$ and $p-p_{mh}$ satisfy:*

$$\begin{array}{cc} & {∥\left(u-u_{mh},b-b_{mh}\right)∥}_{1}\leq C\left(h+H^{2}\right){(∥u∥}_{2}+{∥p∥}_{1}+{∥b∥}_{2})+C∥\left(u_{H}-u_{H}^{m},b_{H}-b_{H}^{m}\right){∥}_{1},\\ & {∥p-p_{mh}∥}_{0}\leq C\left(h+H^{2}\right){(∥u∥}_{2}+{∥p∥}_{1}+{∥b∥}_{2})+C∥\left(u_{H}-u_{H}^{m},b_{H}-b_{H}^{m}\right){∥}_{1}, \end{array}$$

*for all m≥0.*

### 5.3. Two-Level Iterative Method with $\frac{5}{11}<\sigma <1-{\left(\frac{{∥F∥}_{*}}{{∥F∥}_{0}}\right)}^{\frac{1}{2}}$

If $\frac{5}{11}<\sigma <1-{\left(\frac{{∥F∥}_{*}}{{∥F∥}_{0}}\right)}^{\frac{1}{2}}$, we can see that method 3 is the only stable and convergent method. We propose three two-level iterative methods to solve the iterative solution $\left(\left(u_{H}^{m},b_{H}^{m}\right),p_{H}^{m}\right)$ using method 3 on coarse mesh $T_{H}$, and then to solve the correction solution $\left(\left(u_{mh},b_{mh}\right),p_{mh}\right)$ using corrections 1–3 on fine mesh $T_{h}$. Three two-level methods are shown below:

**Step 1**. Solve the MHD equations on the coarse grid, $\left(\left(u_{H}^{m},b_{H}^{m}\right),p_{H}^{m}\right)\in X_{H}\times M_{H}$ provided by method 3.

**Step 2**. One step correction on the fine grid, $\left(\left(u_{mh},b_{mh}\right),p_{mh}\right)\in X_{h}\times M_{h}$ provided by corrections 1–3, respectively.

Then, we further obtain the following theoretical results about the estimation of the above two-level methods.

**Theorem** **13.** Under the conditions of Theorem 4 and the condition $\frac{5}{11}<\sigma <1-{\left(\frac{{∥F∥}_{*}}{{∥F∥}_{0}}\right)}^{\frac{1}{2}}$, $\left(\left(u_{H}^{m},b_{H}^{m}\right),p_{H}^{m}\right)\in X_{H}\times M_{H}$ calculated by method 3, $(\left(u_{mh},b_{mh}\right)$, $p_{mh})$ calculated by correction 1. Then, $\left(u-u_{mh},b-b_{mh}\right)$ and $p-p_{mh}$ satisfy:

$$\begin{array}{c} \begin{array}{cc} {∥\left(u-u_{mh},b-b_{mh}\right)∥}_{1}\leq & C\left(h+H^{2}\right){(∥u∥}_{2}+{∥p∥}_{1}+{∥b∥}_{2})+C∥\left(u_{H}-u_{H}^{m},b_{H}-b_{H}^{m}\right){∥}_{1},\\ {∥p-p_{mh}∥}_{0}\leq C(h+ & H^{2}{)(∥u∥}_{2}+{∥p∥}_{1}+{∥b∥}_{2})+C∥\left(u_{H}-u_{H}^{m},b_{H}-b_{H}^{m}\right){∥}_{1}, \end{array} \end{array}$$

*and $\left(\left(u_{mh},b_{mh}\right),p_{mh}\right)$ calculated by correction 2, $\left(u-u_{mh},b-b_{mh}\right)$ and $p-p_{mh}$ satisfy:*

$$\begin{array}{cc} & {∥\left(u-u_{mh},b-b_{mh}\right)∥}_{1}\leq C\left(h+H^{2}\right){(∥u∥}_{2}+{∥p∥}_{1}+{∥b∥}_{2})+C∥\left(u_{H}-u_{H}^{m},b_{H}-b_{H}^{m}\right){∥}_{1}^{2},\\ & {∥p-p_{mh}∥}_{0}\leq C\left(h+H^{2}\right){(∥u∥}_{2}+{∥p∥}_{1}+{∥b∥}_{2})+C∥\left(u_{H}-u_{H}^{m},b_{H}-b_{H}^{m}\right){∥}_{1}^{2}, \end{array}$$

*and $\left(\left(u_{mh},b_{mh}\right),p_{mh}\right)$ calculated by correction 3, $\left(u-u_{mh},b-b_{mh}\right)$ and $p-p_{mh}$ satisfy:*

$$\begin{array}{cc} & {∥\left(u-u_{mh},b-b_{mh}\right)∥}_{1}\leq C\left(h+H^{2}\right){(∥u∥}_{2}+{∥p∥}_{1}+{∥b∥}_{2})+C∥\left(u_{H}-u_{H}^{m},b_{H}-b_{H}^{m}\right){∥}_{1},\\ & {∥p-p_{mh}∥}_{0}\leq C\left(h+H^{2}\right){(∥u∥}_{2}+{∥p∥}_{1}+{∥b∥}_{2})+C∥\left(u_{H}-u_{H}^{m},b_{H}-b_{H}^{m}\right){∥}_{1}, \end{array}$$

*for all m≥0.*

### 5.4. One-Level Oseen Iterative Method with $1-{\left(\frac{{∥F∥}_{*}}{{∥F∥}_{0}}\right)}^{\frac{1}{2}}<\sigma <1$

It can be analyzed from Theorem 8 that method 3 is the only stable and convergence method with the weaker condition $1-{\left(\frac{{∥F∥}_{*}}{{∥F∥}_{0}}\right)}^{\frac{1}{2}}<\sigma <1$. We present a one-level method to solve the iterative solution $\left(\left(u_{mh},b_{mh}\right),p_{mh}\right)$ on coarse mesh $T_{h}$, as shown below.

**Step 1**. Solve the MHD equations on the fine grid, $\left(\left(u_{mh},b_{mh}\right),p_{mh}\right)\in X_{h}\times M_{h}$ obtained by method 3.

Through the theoretical analysis of Theorem 7 with $1-{\left(\frac{{∥F∥}_{*}}{{∥F∥}_{0}}\right)}^{\frac{1}{2}}<\sigma <1$, the following error estimation results are obtained.

**Theorem** **14.** Assume that $1-{\left(\frac{{∥F∥}_{*}}{{∥F∥}_{0}}\right)}^{\frac{1}{2}}<\sigma <1$. Then, $\left(u_{mh},b_{mh}\right)$ defined by method 3 satisfies

(49) $$\begin{array}{c} {∥\left(u_{mh},b_{mh}\right)∥}_{1}\leq \frac{{∥F∥}_{*}}{\min\left\{R_{e}^{-1},\lambda_{1}R_{m}^{-1}S_{c}\right\}}, \end{array}$$

*and $\left(u_{h}-u_{mh},b_{h}-b_{mh}\right)$ and $p_{h}-p_{mh}$ satisfy:*

(50) $$\begin{array}{cc} & {∥\left(u_{h}-u_{mh},b_{h}-b_{mh}\right)∥}_{1}\leq \sigma^{m}\frac{{∥F∥}_{*}}{\min\left\{R_{e}^{-1},\lambda_{1}R_{m}^{-1}S_{c}\right\}}, \end{array}$$

(51) $$\begin{array}{cc} & {∥p_{h}-p_{mh}∥}_{0}\leq C\sigma^{m}\frac{{∥F∥}_{*}}{\min\left\{R_{e}^{-1},\lambda_{1}R_{m}^{-1}S_{c}\right\}}, \end{array}$$

*for all n≥0.*

Combining Theorems 4 and 14, we further derive the final conclusion of this section

**Theorem** **15.** Assume that $1-{\left(\frac{{∥F∥}_{*}}{{∥F∥}_{0}}\right)}^{\frac{1}{2}}<\sigma <1$, $\left(u-u_{mh},b-b_{mh}\right)$ and $p-p_{mh}$ satisfy

(52) $$\begin{array}{cc} & {∥\left(u-u_{mh},b-b_{mh}\right)∥}_{1}\leq Ch\left({∥u∥}_{2}+{∥b∥}_{2}+{∥p∥}_{1}\right)+\sigma^{m}\frac{{∥F∥}_{*}}{\min\left\{R_{e}^{-1},\lambda_{1}R_{m}^{-1}S_{c}\right\}}, \end{array}$$

(53) $$\begin{array}{cc} & {∥p-p_{mh}∥}_{0}\leq Ch\left({∥u∥}_{2}+{∥b∥}_{2}+{∥p∥}_{1}\right)+C\sigma^{m}\frac{{∥F∥}_{*}}{\min\left\{R_{e}^{-1},\lambda_{1}R_{m}^{-1}S_{c}\right\}}, \end{array}$$

*for all n≥0.*

**Remark** **3.** According to Theorems 9–15, we can find that two-level iterative methods that combine method 1 with correction j (j = 1, 2, 3) have the strongest condition $0<\sigma <\frac{2}{5}$. In addition, if $0<\sigma <\frac{5}{11}$, two-level iterative methods that combine method 2 with correction j (j = 1, 2, 3) are convergence. If $0<\sigma <1-{\left(\frac{{∥F∥}_{*}}{{∥F∥}_{0}}\right)}^{\frac{1}{2}}$, two-level iterative methods that combine method 3 with correction j (j = 1, 2, 3) are convergence. Under the weak condition $1-{\left(\frac{{∥F∥}_{*}}{{∥F∥}_{0}}\right)}^{\frac{1}{2}}<\sigma <1$, the one-level Oseen method is a unique choice.

**Remark** **4.** In terms of the convergence rate, we know that two-level iterative methods that combine method i (i = 1, 2, 3) with correction j (j = 1, 3) are linear convergent. Moreover, two-level methods that combine method i (i = 1, 2, 3) with correction 2 have an exponential convergence rate. Therefore, a two-level method that combined method 2 with correction 2 has a faster convergence speed under the unique condition $0<\sigma <\frac{5}{11}$. In case of $\frac{5}{11}<\sigma <1-{\left(\frac{{∥F∥}_{*}}{{∥F∥}_{0}}\right)}^{\frac{1}{2}}$, a two-level iterative method that combined method 3 with correction 2 converges the fastest.

## 6. Numerical Experiments

In this part, three numerical tests are rendered to substantiate the good performance of our proposed methods for the MHD equations. Taking a fluid problem with smooth true solution and Hartmann flow as examples, the optimal convergence rate and computational cost of the proposed scheme are tested. The last one of the driven cavity flow shows good simulated fluid motion results. Moreover, we use low order nonconforming finite element pair $P_{1}nc$-$P_{1}b$-$P_{1}$ for the velocity, magnetic field and pressure. Throughout this section, we denote M1, M2, M3, C1, C2, and C3 as the abbreviations of methods 1–3 and corrections 1–3.

### 6.1. A Fluid Problem with Smooth True Solution

In this case, a fluid problem with a smooth true solution defined on the domain $\Omega ={\left[0,1\right]}^{2}$ will provide theoretical guidance for the studies and analysis of our proposed methods. This is a very common problem in testing the effectiveness of the proposed methods. The boundary conditions and source terms f, g are established by calculating the analytic solutions, which are shown below:

$$\begin{array}{c} \left\{\begin{array}{c} u\left(x,y\right)=(\alpha \pi {\left(\sin\left(\pi x\right)\right)}^{2}\sin\left(\pi y\right)\cos\left(\pi y\right),-\alpha \pi \sin\left(\pi x\right){\left(\sin\left(\pi y\right)\right)}^{2}\cos\left(\pi x\right)),\\ b(x,y)=(\alpha \sin(\pi x)\cos(\pi y),-\alpha \cos(\pi x)\sin(\pi y)),\\ p(x,y)=\alpha \cos(\pi x)\cos(\pi y). \end{array}\right. \end{array}$$

where α>0 satisfies the uniqueness conditions of two-level methods.

**Figure 1:**
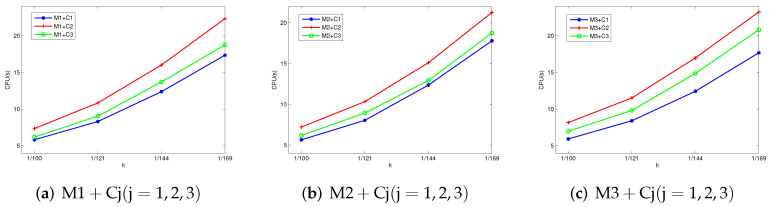
The CPU time for Mi + Cj (i, j = 1, 2, 3) by using $P_{1}nc$ for the velocity field.

Firstly, set $R_{e}=R_{m}=S_{c}=1$. The error values and order of convergence data calculated by our presented two-level methods are shown in Table 1. The CPU times are showed in Figure 1. From this table, we can see that the orders of $∥u-u_{h}^{n}{∥}_{1}$, $∥b-b_{h}^{n}{∥}_{1}$ and $∥p-p_{h}^{n}{∥}_{0}$ are almost all equal to one. It is clear that the two-level algorithms can guarantee the stability of the stabilization method for fluid problems with smooth true solutions. We can see from Table 1 and Figure 1 that Mi + C2 (i = 1, 2, 3) has the best accuracy, while Mi + C1 (i = 1, 2, 3) spends the least computational time with the smaller mesh division. Since the trilinear terms of Mi + C1 (i = 1, 2, 3) are the easiest, the trilinear terms of Mi + C2 (i = 1, 2, 3) are most complicated in our presented two-level algorithms.

Then, numerical results for calculating velocity using the nonconforming element $P_{1}nc$ are given in Table 1, and numerical results for calculating velocity using the piecewise linear element $P_{1}$ are shown in Table 2. By comparing and analyzing the data in Table 1 and Table 2, we can find that the errors calculated by our proposed two-level methods are smaller.

### 6.2. The Hartmann Flow

In this case, we will test a classical MHD problem, the 2D Hartmann flow. It is affected by a steady unidirectional flow in the channel Ω=[0,10]×[−1,1]. Set $Ha=\sqrt{R_{e}R_{m}S_{c}}$ in 2D Hartmann flow. Meanwhile, the transverse magnetic field $b_{D}=\left(0,1\right)$ is inflicted on the boundaries of this system. The analytic solutions are given by:

$$\begin{array}{c} \left\{\begin{array}{c} u(x,y)=(u(y),0),\\ b(x,y)=(B(y),1),\\ p\left(x,y\right)=-Gx-S_{c}B^{2}\left(y\right)/2+p_{0}, \end{array}\right. \end{array}$$

where

$$\begin{array}{c} \begin{array}{c} u\left(y\right)=\frac{GR_{e}}{Ha\cdot \tanh(Ha)}\left(1-\frac{\cosh(yHa)}{\cosh(Ha)}\right),\;B\left(y\right)=\frac{G}{S_{c}}\left(\frac{\sinh(yHa)}{\sinh(Ha)}-y\right). \end{array} \end{array}$$

Furthermore, the boundary conditions are given indicated below:

$$\begin{array}{c} \left\{\begin{array}{c} \left(pI-R_{e}^{-1}\nabla u\right)n=p_{d}n,\;\mathrm{on}\;x=0\;\mathrm{and}\;x=10,\\ u=0,\;\mathrm{on}\;y=\pm 1,\\ n\times b=n\times b_{D},\;\mathrm{on}\;\partial \Omega , \end{array}\right. \end{array}$$

where $p_{d}\left(x,y\right)=p\left(x,y\right)$, $p_{0}$ is zero, and I is the identity matrix.

**Figure 2:**
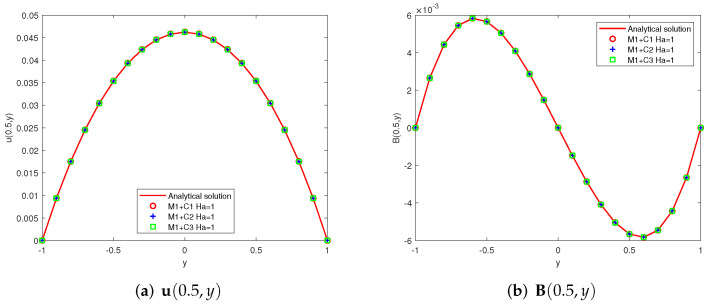
Analytical curves and numerical results obtained by M1 + Cj (j = 1, 2, 3) (x=0.5, −1≤y≤1).

**Figure 3:**
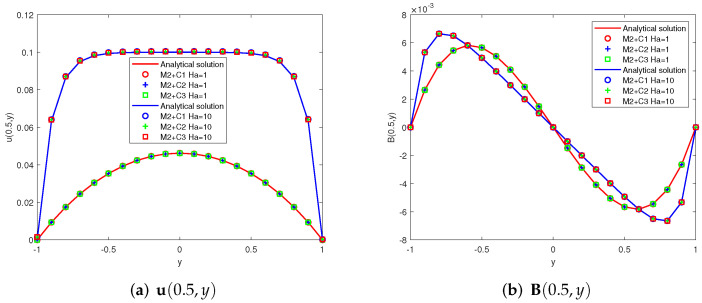
Analytical curves and numerical results obtained by M2 + Cj (j = 1, 2, 3) (x=0.5, −1≤y≤1).

**Figure 4:**
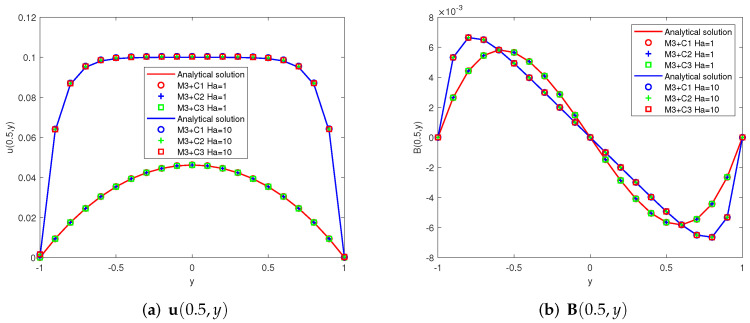
Analytical curves and numerical results obtained by M3 + Cj (j = 1, 2, 3) (x=0.5, −1≤y≤1).

Taking G = 0.1 and considering two different schemes: $\left(1\right)\;Ha=1\;\left(R_{e}=1,R_{m}=1,S_{c}=1\right)$, $\left(2\right)\;Ha=10\;\left(R_{e}=10,R_{m}=1,S_{c}=10\right)$. The first components of the analytic solutions u(y), B(y) and the numerical ones $u(y_{k})$, $B\left(y_{k}\right)\left(y_{k}=-1+0.1k,k=0,\cdots ,20\right)$ provided by Mi + Cj (i, j = 1, 2, 3) are showed in Figure 2, Figure 3 and Figure 4. Since some two-level methods do not converge at high Hartmann numbers, we present only convergent images.

Then, to better illustrate the precision of our proposed algorithms, the error values and order of convergence data calculated by our presented two-level algorithms are shown in Table 3 when scheme (1) is selected. Here, we only show the discretization errors of velocity, magnetic field and pressure of one method, since the numerical results of other methods are roughly the same in numerical values. We can seen from the table that the convergence orders of velocity and magnetic field in $H^{1}$-norm reach the first order, and the pressure in $L^{2}$-norm achieves a better approximate convergence rate higher than the first order. The results show that the two-level algorithms can guarantee the accuracy of numerical experiment for the Hartmann flow problem.

**Figure 5:**
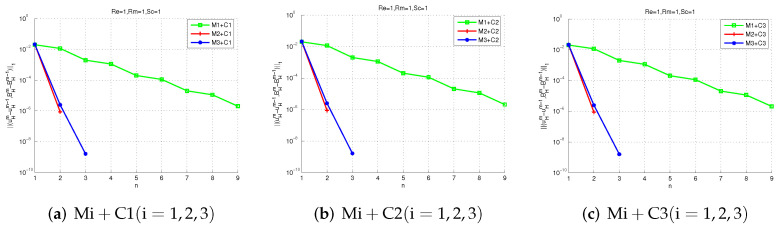
The iteration convergence errors of two-level methods with $R_{e}=1,R_{m}=1,S_{c}=1$.

**Figure 6:**
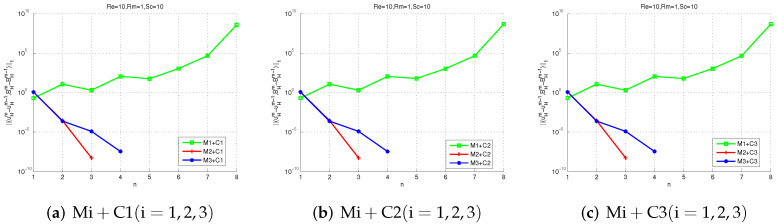
The iteration convergence errors of two-level methods with $R_{e}=10,R_{m}=1,S_{c}=10$.

**Figure 7:**
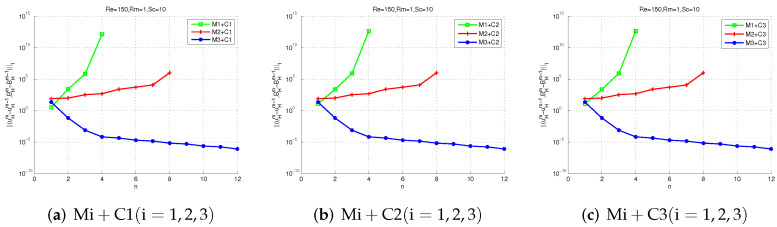
The iteration convergence errors of two-level methods with $R_{e}=150,R_{m}=1,S_{c}=10$.

Next, we test the convergence of Mi + Cj (i, j = 1, 2, 3). Setting G = 0.1 and choosing three different schemes: $\left(1\right)\;Ha=1\;\left(R_{e}=1,R_{m}=1,S_{c}=1\right)$, $\left(2\right)\;Ha=10\;\left(R_{e}=10,R_{m}=1,S_{c}=10\right)$, $\left(3\right)\;Ha=10\sqrt{15}\;\left(R_{e}=150,R_{m}=1,S_{c}=10\right)$. All the nine proposed methods are available for scheme (1) in Figure 5, which means that all the proposed methods are convergent for $0<\sigma <\frac{2}{5}$. However, Figure 6 shows M1 + Cj (j = 1, 2, 3) are not convergent for $\frac{2}{5}<\sigma <\frac{5}{11}$ because the parameter selection is not in $\frac{2}{5}<\sigma <\frac{5}{11}$. Figure 7 shows If $Ha=10\sqrt{15}\;\left(R_{e}=150,R_{m}=1,S_{c}=10\right)$, only M3 + Cj (j = 1, 2, 3) is convergent while Mi + Cj (i = 2, 3, j = 1, 2, 3) is not convergent, since the uniqueness condition is not met.

We can also see from those figures that M1 + Cj (j = 1, 2, 3) only applies to the case of a small Hartmann number. In some cases with large Hartmann numbers, M3 + Cj (j = 1, 2, 3) can be chosen. Figure 5 shows that M2 + Cj (j = 1, 2, 3) converges faster than Mi+Cj (i = 1, 2, j = 1, 2, 3) since M2 has exponential convergence. All in all, these numerical experimental results demonstrate the effectiveness of our theoretical analysis and the proposed methods.

### 6.3. Driven Cavity Flow

In the last case, the numerical simulation of a classical fluid problem with driven cavity flow is showed. We consider the flow in the 2D domain Ω=[−1,1]×[−1,1] with $\Gamma_{D}=\partial \Omega$, and there is no analytical solution. Let the external force terms f and g be zero; then, their boundary conditions are defined as follows:

$$\begin{array}{c} \left\{\begin{array}{c} u(x,y)=0,\;\mathrm{on}\;x=\pm 1\;\mathrm{and}\;y=-1,\\ u(x,y)=(1,0),\;\mathrm{on}\;y=1,\\ n\times b=n\times b_{D},\;\mathrm{on}\;\partial \Omega , \end{array}\right. \end{array}$$

where $b_{D}=\left(1,0\right)$.

**Figure 8:**
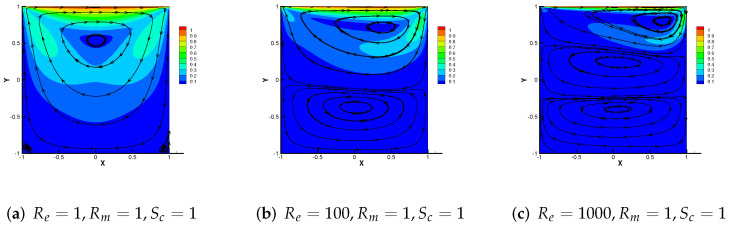
Numerical streamlines of the velocity drawn using data obtained from M3 + C2, wherein $R_{e}$ is set as 1, 100 and 1000 respectively.

**Figure 9:**
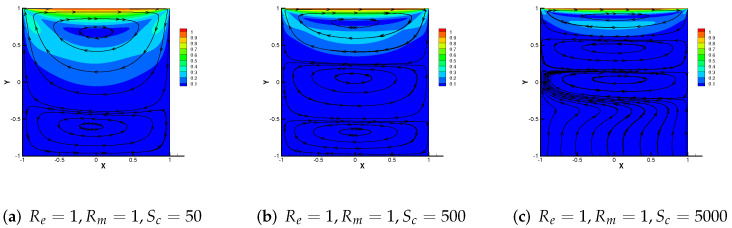
Numerical streamlines of the velocity drawn using data obtained from M3 + C2, wherein $S_{c}$ is set as 50, 500 and 5000 respectively.

From the above two examples and theoretical analysis, M3 + Cj (j = 1, 2, 3) has a wider application. Therefore, we mainly simulate the effect of the driven cavity flow by M3 + C2 in this part. In Figure 8, the velocity streamline for three different parameters $R_{e}$ = 1, 100 and 1000 with $R_{m}$ = 1, $S_{c}$ = 1 are showed. As $R_{e}$ enhances, the number of vortices produced by velocity streamlines increases to three. We use the same change pattern in Figure 9, and the streamlines of velocity for $S_{c}$= 50, 500, 5000 with $R_{e}$ = 1, $R_{m}$ = 1, from which we can notice that the vortices produced by velocity streamlines also divide into three vortices and move upward.

**Figure 10:**
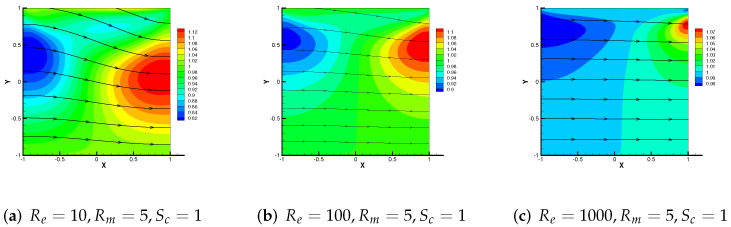
Numerical streamlines of the magnetic field drawn using data obtained from M3 + C2, wherein $R_{e}$ is set as 10, 100 and 1000 respectively.

**Figure 11:**
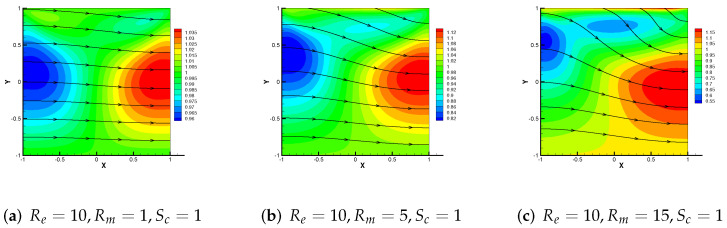
Numerical streamlines of the magnetic field drawn using data obtained from M3 + C2, wherein $R_{m}$ is set as 1, 5 and 15 respectively.

**Figure 12:**
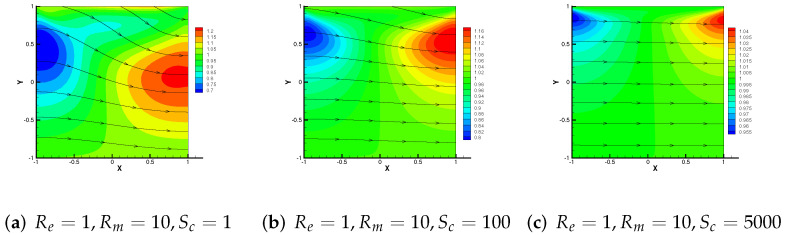
Numerical streamlines of the magnetic field drawn using data obtained from M3 + C2, wherein $S_{c}$ is set as 1, 100 and 5000 respectively.

Then, Figure 10, Figure 11 and Figure 12 show the variation trend of numerical streamlines of the magnetic by M3 + C2 as parameters change. We vary $R_{e}$ = 10, 100, 1000 with $R_{m}$ = 5, $S_{c}$ = 1 in Figure 10. Analogously, we vary $S_{c}$ from 1 in (a), to 100 in (b), then to 5000 in (c) with $R_{e}$ = 1, $R_{m}$ = 10 in Figure 12, from which we can all observe that the streamlines have a tendency to change straight.

Conclusively, Figure 11 shows the trends of magnetic field streamlines for $R_{m}$ = 1, 5, 15 with $R_{e}$ = 10, $S_{c}$ = 1. As $R_{m}$ enhances, the shape of streamline changes from a straight line to a curve. The above phenomenon indicates an increase in curvature.

## 7. Conclusions

Based on nonconforming FEM, several two-level methods for solving the stationary incompressible MHD equations have been presented under different unique conditions in this paper. Combining theoretical analysis with numerical experiments, the two-level method that combined method 2 with correction 2 has faster convergence speed and better calculation accuracy under the unique condition $0<\sigma <\frac{5}{11}$. In case of $\frac{5}{11}<\sigma <1-{\left(\frac{{∥F∥}_{*}}{{∥F∥}_{0}}\right)}^{\frac{1}{2}}$, the two-level method that combined method 3 with correction 2 has a good advantage. If $1-{\left(\frac{{∥F∥}_{*}}{{∥F∥}_{0}}\right)}^{\frac{1}{2}}<\sigma <1$, one-level Oseen iterative method is a unique scheme.

## Figures and Tables

**Table 1:** Errors obtained from Mi + Cj (i, j = 1, 2, 3) by using $P_{1}nc$ for the velocity field.

Method	*H*	*h*	$∥u-u_{h}^{n}{∥}_{1}$	Order	$∥b-b_{h}^{n}{∥}_{1}$	Order	$∥p-p_{h}^{n}{∥}_{0}$	Order
Mi+C1	$\frac{1}{2}$	$\frac{1}{4}$	5.11 × 10${}^{-1}$	∖	3.58 × 10${}^{-1}$	∖	2.84 × 10${}^{0}$	∖
Mi+C2	$\frac{1}{2}$	$\frac{1}{4}$	5.10 × 10${}^{-1}$	∖	3.57 × 10${}^{-1}$	∖	2.73 × 10${}^{0}$	∖
Mi+C3	$\frac{1}{2}$	$\frac{1}{4}$	5.12 × 10${}^{-1}$	∖	3.56 × 10${}^{-1}$	∖	2.81 × 10${}^{0}$	∖
Mi+C1	$\frac{1}{4}$	$\frac{1}{16}$	1.38 × 10${}^{-1}$	0.94	9.70 × 10${}^{-2}$	0.94	2.47 × 10${}^{-1}$	1.76
Mi+C2	$\frac{1}{4}$	$\frac{1}{16}$	1.38 × 10${}^{-1}$	0.94	9.29 × 10${}^{-2}$	0.97	1.83 × 10${}^{-1}$	1.95
Mi+C3	$\frac{1}{4}$	$\frac{1}{16}$	1.38 × 10${}^{-1}$	0.95	9.36 × 10${}^{-2}$	0.99	2.46 × 10${}^{-1}$	1.76
Mi+C1	$\frac{1}{8}$	$\frac{1}{64}$	3.47 × 10${}^{-2}$	1.00	2.52 × 10${}^{-2}$	0.97	5.30 × 10${}^{-2}$	1.11
Mi+C2	$\frac{1}{8}$	$\frac{1}{64}$	3.47 × 10${}^{-2}$	1.00	2.33 × 10${}^{-2}$	1.00	2.21 × 10${}^{-2}$	1.53
Mi+C3	$\frac{1}{8}$	$\frac{1}{64}$	3.47 × 10${}^{-2}$	1.00	2.35 × 10${}^{-2}$	1.00	5.96 × 10${}^{-2}$	1.02
Mi+C1	$\frac{1}{16}$	$\frac{1}{256}$	8.69 × 10${}^{-3}$	1.00	6.39 × 10${}^{-3}$	0.96	1.30 × 10${}^{-2}$	1.01
Mi+C2	$\frac{1}{16}$	$\frac{1}{256}$	8.68 × 10${}^{-3}$	1.00	5.82 × 10${}^{-3}$	1.00	2.75 × 10${}^{-3}$	1.50
Mi+C3	$\frac{1}{16}$	$\frac{1}{256}$	8.69 × 10${}^{-3}$	1.00	5.89 × 10${}^{-3}$	1.00	1.54 × 10${}^{-2}$	0.98

**Table 2:** Errors obtained from Mi + Cj (i, j = 1, 2, 3) by using $P_{1}b$ for the velocity field.

Method	*H*	*h*	$∥u-u_{h}^{n}{∥}_{1}$	Order	$∥b-b_{h}^{n}{∥}_{1}$	Order	$∥p-p_{h}^{n}{∥}_{0}$	Order
Mi+C1	$\frac{1}{2}$	$\frac{1}{4}$	5.68 × 10${}^{-1}$	∖	3.56 × 10${}^{-1}$	∖	6.21 × 10${}^{0}$	∖
Mi+C2	$\frac{1}{2}$	$\frac{1}{4}$	5.68 × 10${}^{-1}$	∖	3.56 × 10${}^{-1}$	∖	6.21 × 10${}^{0}$	∖
Mi+C3	$\frac{1}{2}$	$\frac{1}{4}$	5.68 × 10${}^{-1}$	∖	3.56 × 10${}^{-1}$	∖	6.22 × 10${}^{0}$	∖
Mi+C1	$\frac{1}{4}$	$\frac{1}{16}$	1.52 × 10${}^{-1}$	0.95	9.34 × 10${}^{-2}$	0.97	7.66 × 10${}^{-1}$	1.51
Mi+C2	$\frac{1}{4}$	$\frac{1}{16}$	1.52 × 10${}^{-1}$	0.95	9.29 × 10${}^{-2}$	0.97	6.57 × 10${}^{-1}$	1.62
Mi+C3	$\frac{1}{4}$	$\frac{1}{16}$	1.52 × 10${}^{-1}$	0.95	9.36 × 10${}^{-2}$	0.96	9.18 × 10${}^{-1}$	1.38
Mi+C1	$\frac{1}{8}$	$\frac{1}{64}$	3.79 × 10${}^{-2}$	1.00	2.35 × 10${}^{-2}$	0.99	1.38 × 10${}^{-1}$	1.23
Mi+C2	$\frac{1}{8}$	$\frac{1}{64}$	3.78 × 10${}^{-2}$	1.00	2.33 × 10${}^{-2}$	1.00	7.41 × 10${}^{-2}$	1.57
Mi+C3	$\frac{1}{8}$	$\frac{1}{64}$	3.79 × 10${}^{-2}$	1.00	2.38 × 10${}^{-2}$	0.99	2.36 × 10${}^{-1}$	0.98
Mi+C1	$\frac{1}{16}$	$\frac{1}{256}$	9.46 × 10${}^{-3}$	1.00	5.88 × 10${}^{-3}$	1.00	3.12 × 10${}^{-2}$	1.07
Mi+C2	$\frac{1}{16}$	$\frac{1}{256}$	9.44 × 10${}^{-3}$	1.00	5.82 × 10${}^{-3}$	1.00	8.98 × 10${}^{-3}$	1.52
Mi+C3	$\frac{1}{16}$	$\frac{1}{256}$	9.47 × 10${}^{-3}$	1.00	5.97 × 10${}^{-3}$	1.00	6.12 × 10${}^{-2}$	0.98

**Table 3:** Errors obtained from M2 + C2.

H	h	$∥u-u_{h}^{n}{∥}_{1}$	Order	$∥b-b_{h}^{n}{∥}_{1}$	Order	$∥p-p_{h}^{n}{∥}_{0}$	Order
$\frac{1}{4}$	$\frac{1}{16}$	6.38 × 10${}^{-2}$	∖	6.63 × 10${}^{-2}$	∖	2.38 × 10${}^{-4}$	∖
$\frac{1}{5}$	$\frac{1}{25}$	4.09 × 10${}^{-2}$	1.00	4.24 × 10${}^{-2}$	1.00	1.22 × 10${}^{-4}$	1.49
$\frac{1}{6}$	$\frac{1}{36}$	2.84 × 10${}^{-2}$	1.00	2.95 × 10${}^{-2}$	1.00	7.09 × 10${}^{-5}$	1.49
$\frac{1}{7}$	$\frac{1}{49}$	2.09 × 10${}^{-2}$	1.00	2.16 × 10${}^{-2}$	1.00	4.47 × 10${}^{-5}$	1.50
